# Genome wide screening of RNAi factors of *Sf21* cells reveal several novel pathway associated proteins

**DOI:** 10.1186/1471-2164-15-775

**Published:** 2014-09-09

**Authors:** Subhanita Ghosh, Pavan Kumar Kakumani, Ajit Kumar, Pawan Malhotra, Sunil K Mukherjee, Raj K Bhatnagar

**Affiliations:** Insect Resistance Group, International Centre for Genetic Engineering and Biotechnology, Aruna Asaf Ali Marg, New Delhi, 110067 India; Centre for Bioinformatics, M.D. University, Rohtak, 124001 India; Malaria Group, International Centre for Genetic Engineering and Biotechnology, Aruna Asaf Ali Marg, New Delhi, 110067 India; Department of Genetics, University of Delhi South Campus, Benito Juarez Road, New Delhi, 110021 India

**Keywords:** RNA interference, siRNA screening, *Sf21* cells, Genome-wide screening, Insect RNAi, *Spodoptera frugiperda*

## Abstract

**Background:**

RNA interference (RNAi) leads to sequence specific knock-down of gene expression and has emerged as an important tool to analyse gene functions, pathway analysis and gene therapy. Although RNAi is a conserved cellular process involving common elements and factors, species-specific differences have been observed among different eukaryotes. Identification of components for RNAi pathway is pursued intensively and successful genome-wide screens have been performed for components of RNAi pathways in various organisms. Functional comparative genomics analysis offers evolutionary insight that forms basis of discoveries of novel RNAi-factors within related organisms. Keeping in view the academic and commercial utility of insect derived cell-line from *Spodoptera frugiperda*, we pursued the identification and functional analysis of components of RNAi-machinery of *Sf21* cell-line using genome-wide application.

**Results:**

The genome and transcriptome of *Sf21* was assembled and annotated. *In silico* application of comparative genome analysis among insects allowed us to identify several RNAi factors in *Sf21* line. The candidate RNAi factors from assembled genome were validated by knockdown analysis of candidate factors using the siRNA screens on the *Sf21-gfp* reporter cell-line. Forty two (42) potential factors were identified using the cell based assay. These include core RNAi elements including Dicer-2, Argonaute-1, Drosha, Aubergine and auxiliary modules like chromatin factors, RNA helicases, RNA processing module, signalling allied proteins and others. Phylogenetic analyses and domain architecture revealed that *Spodoptera frugiperda* homologs retained identity with Lepidoptera (*Bombyx mori*) or Coleoptera (*Tribolium castaneum*) sustaining an evolutionary conserved scaffold in post-transcriptional gene silencing paradigm within insects.

**Conclusion:**

The database of RNAi-factors generated by whole genome association survey offers comprehensive outlook about conservation as well as specific differences of the proteins of RNAi machinery. Understanding the interior involved in different phases of gene silencing also offers impending tool for RNAi-based applications.

**Electronic supplementary material:**

The online version of this article (doi:10.1186/1471-2164-15-775) contains supplementary material, which is available to authorized users.

## Background

RNA interference is an evolutionarily conserved gene-regulatory mechanism for host defence against invading genetic elements. RNAi activity is present in a wide variety of eukaryotic organisms [1–4] and is triggered by the presence of double-stranded RNA (dsRNA) or small interfering RNAs (siRNAs), resulting in the degradation or inactivation of cognate messenger RNA [5, 6]. RNAi thus plays important roles in a broad variety of biological pathways such as antiviral defence, epigenetic regulation, DNA elimination and heterochromatin formation etc [7–9]. Biochemical and structural biology studies have provided insights into the molecular details of RNAi and have shown that RNAi is a two-step process in simplistic terms. The first step, referred to as initiation, involves cleavage of dsRNA into small interfering RNAs of ~21-25nt size with two-nucleotide, 3′ overhanging ends [10, 11]. This step is governed in ATP dependent manner by RNase III class of protein(s) called Dicer(s) and few cognate RNA binding protein(s) such as TRBP or R2D2 [12, 13]. The second step, referred to as ‘Slicing’, is the main pivotal degradation step where siRNAs join a multi-nuclease complex called RISC complex, where the endonuclease degrades the single stranded messenger RNA in a site-specific manner. Slicer activity is guided by Argonaute-2, an RNAase H class of enzyme that has been shown to be associated with FMR, Vig and Tudor-SN proteins [14–16]. Besides these basic factors, other accessory factors also have been shown to participate in the RNAi process [17–19]. However, little is known about RNAi intermediates, RNA-protein complexes and mechanisms of formation of different complexes.

Upon completion of whole genome sequences for a number of organisms, a limited number of reports are available for genome-wide screening of components of RNAi pathways. The first genome-wide screen for the identification of genetic components of RNAi was performed using an engineered RNAi sensor strain of *Caenorhabditis elegans*[20]. The RNAi screen identified about 90 genes which included 11 well known components of RNAi machinery, RNA binding/processing factors, chromatin associated factors, DNA recombination proteins and nuclear import/export proteins etc. The protein-protein interaction maps of these proteins suggested links between germ line and somatic gene silencing as well as RNA-dependent gene regulatory pathways. Later, a couple of studies carried-out genome-wide RNAi screen in *Drosophila* cell lines and identified seven known RNAi genes (*Ago-2*, *Tis11*, *Hsc70-3*, *Hsc70-4* and *hdc*) and two annotated genes (*CG17265* and *CG10883*) [21]. RNAi has been successfully used in functional genomic studies in many living insect groups such as Coleoptera, Diptera, Dictyoptera, Hemiptera, Hymenoptera, Isoptera, Lepidoptera, Neuroptera and Orthoptera [22–28]. Silencing efficiency has been shown to vary between various insect species and few of these insects are refractory to robust systemic RNAi effects. To understand the differences in the processes of RNAi among various insect species, it is important to carry-out genome-wide RNAi analysis among different insect species. Up till now such a genome-wide RNAi analysis has been performed *in vivo* in *Tribolium castaneum, Nilaparvata lugens* and *in silico* in *Bombyx mori*. Many of these RNAi factors have been revealed functionally by administration of respective dsRNAs [27, 29]. In the present study; we carried-out genome-wide analysis of the RNAi components in insect *Spodoptera frugiperda* (*Sf21*) cell line using both the bioinformatic and the experimental validation approaches.

*Sf21* cell line is originally derived from one of the most agronomically important polyphagous pest *Spodoptera* and is permissive to multiple virus infection. We have successfully used *Sf21*cells for the functional validation of number of RNAi suppressor proteins [30–32]. Recently, we have assembled the complete genome and transcriptome sequence of *Sf21* cell line. Using information from both the genome and transcriptome data, we comprehensively investigated the repertoire of genes involved in RNAi in *Sf21* cell line by comparing the sequence data with orthologues from *Bombyx mori*, *Drosophila melanogaster*, *Tribolium castaneum* and *C. elegans.* Role of these putative RNAi genes was further confirmed using an insect cell-line expressing *gfp* reporter, developed in our laboratory [30]. An evolutionary conservation of core RNAi gene set was observed. However, a few new RNAi effector components specific to *Spodoptera sp.* were also identified. We applied available database of the RNAi-factors from different insects to generate comparative profile of *Sf21* based RNAi components with other phylogenetically distinct insects, thus providing insight into diversity of RNAi factors.

## Results

### *In silico*identification of RNAi factors

To perform a genome-wide analysis of RNAi components in *Sf21*, a genomic library was generated for *Sf21* cells and sequenced on Illumina platform. Additionally, genome-wide transcriptome analysis for *Sf21* cell line was also accomplished. The *Sf21* genome was assembled using Velvet and gene prediction analysis was performed using Augustus. The transcriptome assembly was done using both Velvet and Oases and corresponding ORF sequences were identified using EMBOSS with default parameters [33] (SUB620801). The ORF sequences corresponding to RNAi factors were predicted with the help of UniProt data set for invertebrates and also using sequences of RNAi factors identified in *Caenorhabditis elegans*, *Tribolium castaneum* and *Drosophila melanogaster* genomes as the guide orthologs. By Blast search, we identified a total of 80 potential RNAi factors from the mined genome and transcriptome dataset of *Sf21* cells and these factors were evaluated for validation using three factor-specific siRNAs that were transfected in the *Sf21-gfp* reporter cell line for *gfp* reversion [30].

### *In vivo*validation of putative *Sf21*RNAi candidates by reporter based siRNA screen

We have been using *gfp* expressing *Sf21* cell line for the functional genomic studies as well as to understand host-parasite interactions [30, 34]. The RNAi screen for the putative eighty *Sf21* RNAi factors was carried out using *gfp* fluorescent *Sf21* cell line. At least three siRNAs were designed and tested for each of the eighty *Sf21* RNAi factors (Additional file 1). Each of these siRNAs was co-transfected with *gfp* siRNA in the stably *gfp* expressing *Sf21* cell line. *Gfp* fluorescence was monitored by FACS analysis as well as by microscopic examination. The putative siRNAs that were able to restore the *gfp* fluorescence of the silenced line were analysed and their corresponding genes/proteins were considered as the true RNAi factors (Table 1). The knock down efficiency of each siRNAs specific to putative candidates has been determined a-priori by performing quantitative Real-Time PCR experiment before using these for *gfp*-reversion experiment. We show the efficacies of a few representative siRNAs in Additional file 2. These siRNAs targeted three genes, namely, Dcr-1, Ago-1 and Drosha for which *gfp* reversion was scored well and also another three genes, namely, Loquacious, Tudor and Sil-2, which failed to show low or no reversion.

**Table 1:** **Putative RNAi factors of** ***Sf21*** **quantified by** ***gfp*** **reversion assay**

Gene	% ***gfp*** reversion	Identity %		
		Bm	Tc	Dm
**Core RNAi factors**				
Argonaute-1	48.6	94	88	83
Argonaute-3	12.25	69	44	43
Dicer-2	43.61	64	38	30
Dicer-1	25.43	68	43	40
Aubergine	41.42	69	49	44
Drosha	39.97	84	66	60
Pasha	45.56	69	53	38
Loquacious	16.56	84	66	56
R2D2	23.74	60	28	30
**Auxilliary RNAi factors**				
**DEAD RNA helicase domain proteins**				
Dbp45A subfamily	24.22	87	56	57
VASA subfamily	31.08	80	59	58
DDX18/HAS1 subfamily	61.54	74	53	52
**RNA processing module**				
U1A snRNP	36.87	90	83	73
SmG	23.79	94	83	77
Integrator complex subunit (Int11)	76.11	99	92	91
Zn finger protein	38.7	69	35	33
Regulator of nonsense transcripts 1 homolog (smg-2 like)	11.45	97	81	76
**Protein Kinase family of signalling allied components**				
CaM Kinase	25.86	96	84	83
Serine/threonine p21-activated kinase (PAK) mbt like protein	31.32	86	62	79
cAMP-dependent protein kinase C1	39.3	99	98	95
Protein Kinase C	21.58	97	92	87
IKK-beta	30.39	66	36	30
STE20/Fray	40.41	86	75	73
MAPKK4	39.96	90	75	73
**Multi drug resistance cassette transporter**				
MDR1A	67.51	73	51	46
**Tudor domain protein**				
Tudor**	-	79	56	54
**Sid like protein**				
Sil-2	9.23	65	33	
**Chromatin factors**				
Histone3 Lysine4 N-methyltransferase	38.57	82	45	60
Histone deacetylase 3 like	26.71	94	85	79
Gas41	26.92	88	71	70
**Translational unit**				
eIF2B-gamma	30.55	79	48	41
eIF4AII	24.16	96	83	80
eIF4AIII	30.92	99	93	96
RPL23P	28.09	98	93	84
**Cell division associated elements**				
KIF18A-like	24.97	76	63	59
Cyclin-dependent kinase 5 homolog	23.61	99	91	80
KIF3A-like	44.75	96	58	69
**Metabolic factor**				
Isocitrate dehydrogenase [NAD] subunit alpha	67.32	82	79	72
**Others**				
Myosin VIIa-like	26.44	95	88	84
Nucleolar complex protein 2 homolog	35.81	69	53	55
WD 40 like repeat domain	30.55	97	94	90
S-phase kinase-associated protein (SkpA)	35.7	98	93	83

The table contains 42 *Sf21* RNAi factors with their respective percentage of *gfp* reversion in the functional assay. Comparative genomics analyses for identity of each *Sf21* candidate with other insects *Bombyx mori* (Bm), *Tribolium castaneum* (Tc) and *Drosophila melanogaster* have been done by homology based search.

**Several RNAi related studies reveal Tudor as one of the conventional RNAi factor. Although reversion assay did not show expected outcome in our study; we consider Tudor as one of the potential RNAi candidate thus mentioning 42 RNAi candidates in the present study.

**Figure 1:**
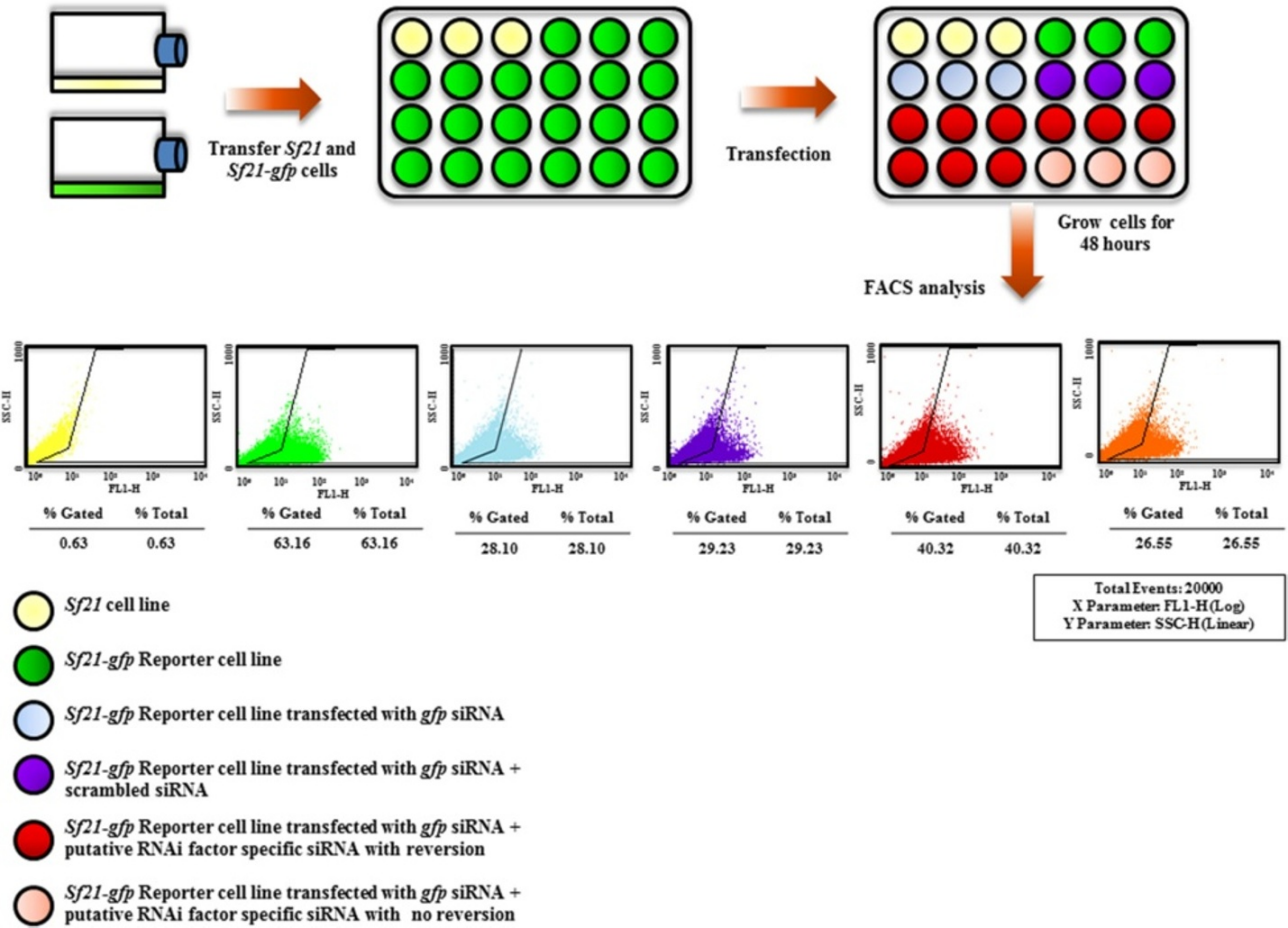
**A genome-wide screen for identification of** ***Sf21*** **RNAi factors.** Schematic representation of experimental design of the siRNA mediated reversion assay in *Sf21*cell line for RNAi screening.

**Figure 2:**
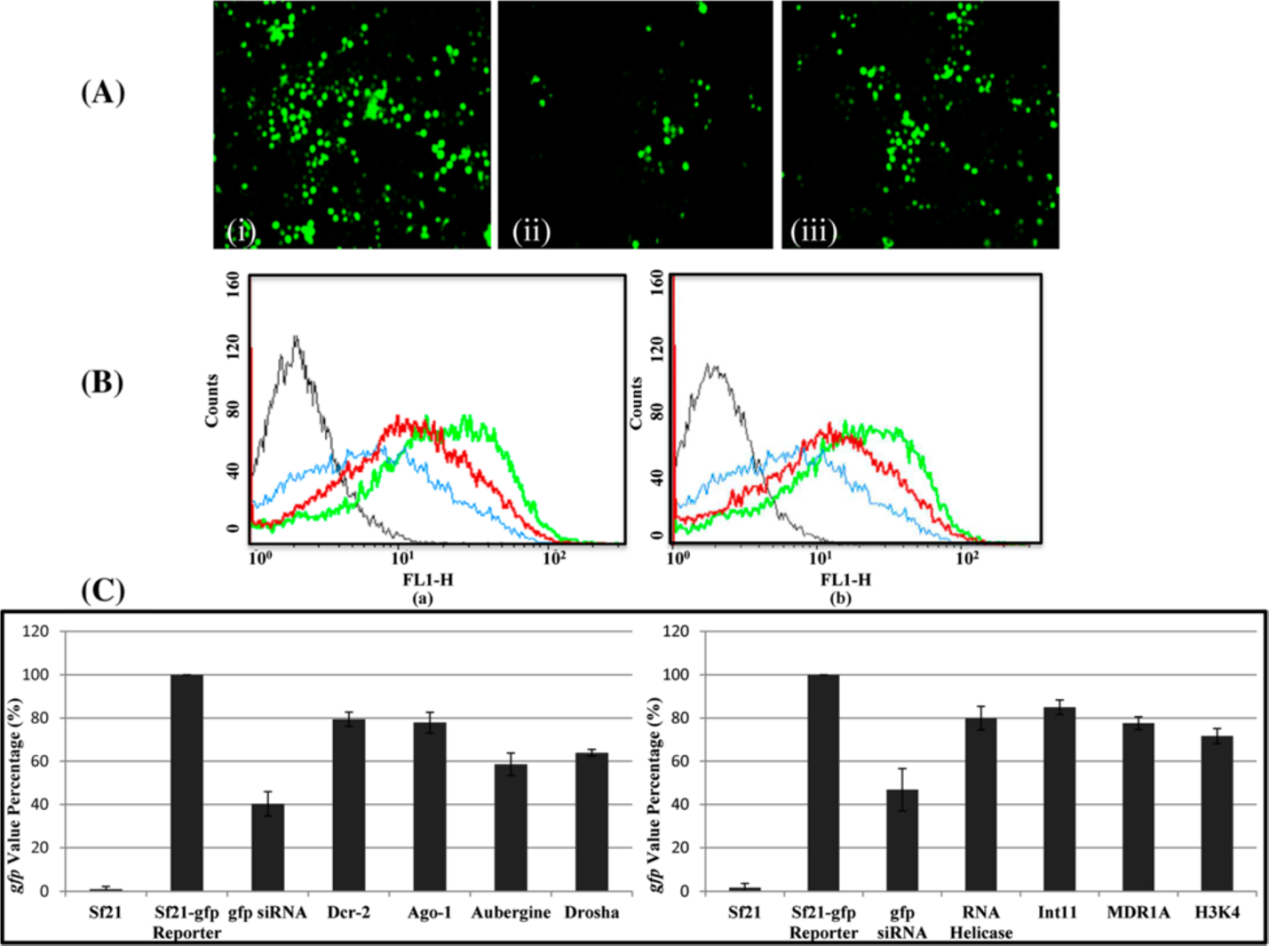
**Reversion Assay for putative RNAi factor. (A)** (i) Fluorescence imaging of *Sf21-gfp* reporter cell line (ii) *Sf21-gfp* reporter cell line transfected with *gfp* siRNA (iii) *Sf21-gfp* reporter cell line co-transfected with putative RNAi factor Sf-Dcr-2 specific siRNA with *gfp* siRNA. **(B)** Histogram overlay plot shows different *gfp* expression in transfected *Sf21* cell line. Plots depict number of cells (counts) on y axis vs. expression of the *gfp* reporter (FLH-1) on x axis. Black trace, normal *Sf21* cells; green trace, *Sf21-gfp* reporter cell line; blue trace, siRNA transfected *gfp* silenced *Sf21-gfp* cells; red trace, *gfp* reverted *Sf21-gfp* cells with putative RNAi factor transfection (a) Sf-Ago-1 (b) Sf-Dcr-2. **(C)** The bar graph representation of the FACS results for (i) putative core RNAi factors Dicer-2, Argonaute-1, Aubergine, Drosha and (ii) auxiliary RNAi candidates RNA helicase DDX18/HAS1 subfamily, Integrator complex subunit (Int11), Multi Drug Resistance MDR1A transporter and Histone-3 Lysine-4 N-Methyltransferase in *Sf21-gfp* reporter cell line represented on the x axis, with the percentage of cells expressing *gfp* on the y axis. Data shown are mean ± SD of three independent experiments.

The schematic of *gfp* reversion assay for identification of putative RNAi candidates in *Sf21* has been shown in Figure 1. Figure 2A shows the reversion in *gfp* expression with siRNA corresponding to putative Dcr-2 gene by microscopic examination. Figure 2B shows quantitative measurement of *gfp* fluorescence by FACS analysis in lines transfected with siRNAs corresponding to putative Dcr-2 as well as Ago-1 genes. Each of the siRNA transfection experiments were carried out in triplicate and the number of fluorescent cells was recorded from the FACS data. The average number of *gfp* expressing cells measured in this way has been displayed in Figure 2C. Figure 2C shows the bar graph with ± SD values showing the reversion in *gfp* expression for few core and accessory RNAi factors. Following identical regimen and protocol, in total forty two candidate RNAi factors were validated from a pool of 80 potential candidates.

The experiments were carried out in several replicates so that the data could be statistically valid. However, the variations amongst the replicates were statistically insignificant. For calculating the *gfp*-reversion values, we have used the value for the particular siRNA that showed maximum reversion within the set of three siRNAs. The particular siRNA was then transfected three times independently for the reversion experiments and the average value of these replicates was reported accordingly. Additional file 3 shows % of *gfp* quantification from post transfection FACS result of the functional assay for all three sets of siRNAs from each of a few selected representative candidate genes. These genes include core RNAi factors like Dcr-2, Ago-1, Drosha, Pasha, Aubergine, Loquacious which have shown reversion of *gfp* and others including Auxilliary RNAi factors, like DDX18/HAS1 subfamily RNA helicase, Multi Drug Resistance (MDR1A), Isocitrate Dehydrogenase, Tudor, Sil-2. The table also contains some genes from 80 putative candidates, namely, Serine/threonine protein phosphatase 2A, Myosin-XV -like and Splicing factor 3 subunit 1. Negligible or mild range of *gfp* reversion was scored with the latter genes.

These genes were further classified based on their perspective role as Core and Auxiliary RNAi components. Dicer, Argonaute, microprocessor complex (Drosha/Pasha), Aubergine, R2D2 and Loquacious were regarded as integral units of RNAi machinery. The auxiliary components comprised of various subcellular regulatory moieties such as DEAD RNA helicase, RNA processing module, chromatin factors, translational machinery, cell division/cell cycle associated elements, protein kinases, signalling allied components, Sid-like proteins and others as part of RNAi-effector complex [20, 29]. These sequences were further analysed to study the domain organization and ancestral relation with their counterparts from other insects, like *B. mori*, *T. castenum* and *D. melanogaster*.

### *Spodoptera frugiperda*Core RNAi factors

#### Ribonuclease III family and dsRBM-containing proteins

Dicer proteins belong to RNase III family of bidentate ribonuclease that selectively cleaves double-stranded RNA precursors with 5′-terminal phosphate and a 2-nucleotide 3′ overhang to produce ~21 nucleotides small interfering RNA [10, 35]. These proteins have several conserved domains: two amino terminal DexH-Box helicase domains, a PAZ (Piwi/Zwille) domain, two tandem RNase III domains and carboxy-terminal dsRNA binding domain(s) [36, 37]. Number of Dicer family of proteins varies in different organisms. A single Dicer protein is involved in both the siRNA and miRNA pathways in *C. elegans* and mammals [20, 38], while *Drosophila*, the prime insect model organism and Coleoptera *Tribolium*; have two Dicer enzymes each; Dcr-1 and Dcr-2. Dm-Dcr-1, which lacks an amino-terminal helicase domain but has a PAZ domain, is involved in the miRNA pathway, while Dm-Dcr-2 lacks PAZ domain but has amino-terminal helicase domain and is involved in siRNA pathway [39]. However, only Bm-Dcr-2 has been annotated yet from Silk worm *Bombyx mori* genome [40].

**Figure 3:**
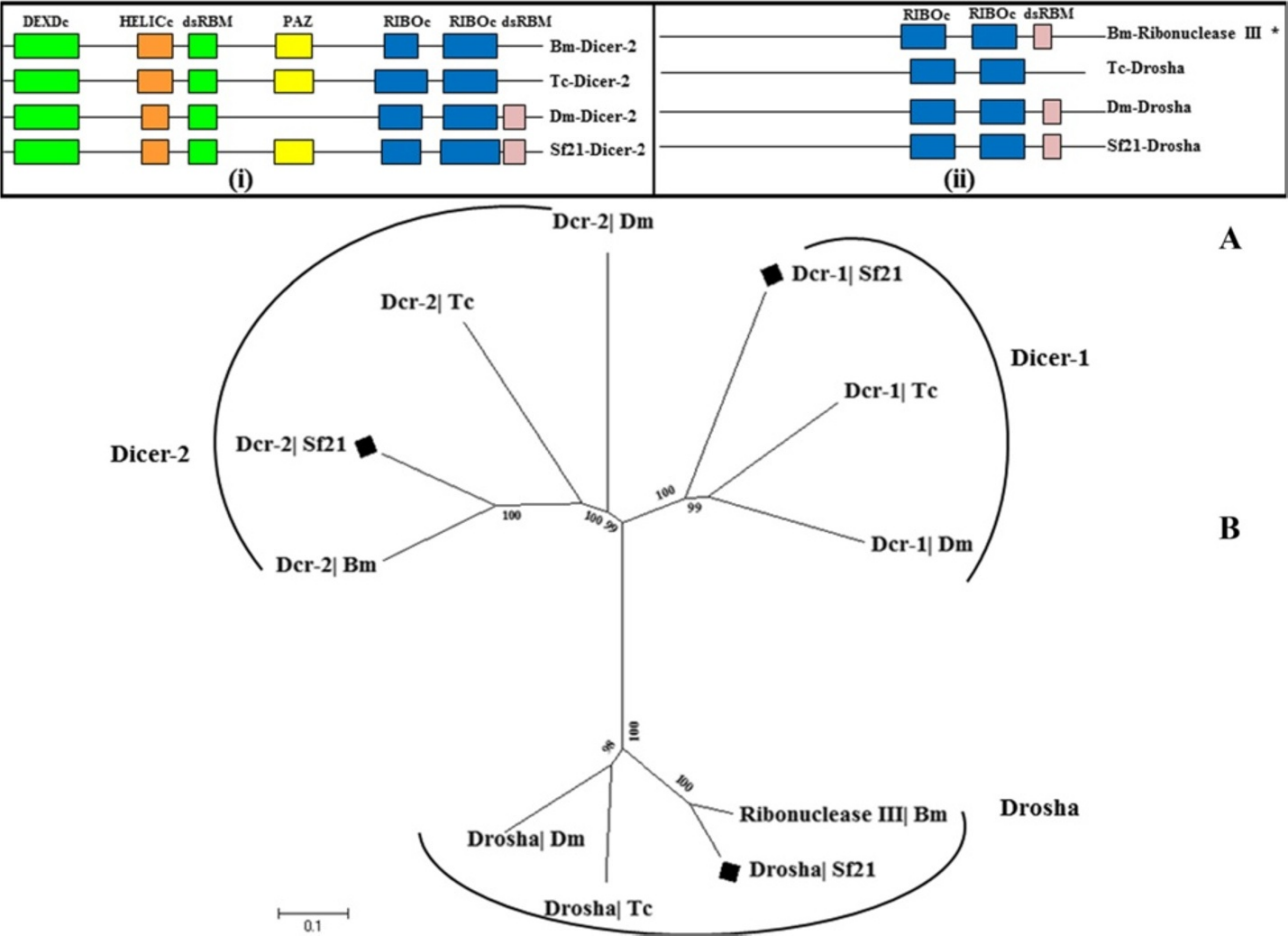
**Domain analysis and phylogenetic tree of Dicer and Ribonuclease III protein family. (A)** (i) Domain organisation clearly indicates the presence of an amino terminal DEAD domain with significant similarity with other Dicer homologs of *Bombyx*, *Tribolium* and *Drosophila* that helps to classify conservation of *Sf21* RNAi factor *Dicer-2* gene. (ii) The other Ribonuclease III component Drosha of *Tribolium* is devoid of N-terminal dsRBM. While Sf21-Drosha shows similarity with *Drosophila* and *Bombyx*. **(B)** Neighbor-joining tree is based on CLUSTALW alignment of full length proteins Dicer-1, Dicer-2 and Drosha as an out group. The phylogenetic tree distributes three different clusters where Sf-Dicer-2 is in the same clade with Bm-Dicer-2 and Sf-Drosha with a Bm-Ribonuclease III. (*) indicates a Bm-Ribonuclease III as a putative Drosha.

We identified two Dicer genes Sf-Dcr-1 and Sf-Dcr-2 from *Sf21* genome data. Domain architecture of Sf-Dcr-2 exhibits the unique pattern with ATP-dependent DEAD RNA Helicase/ PAZ/ Ribonuclease III/ dsRBM, resembling other insect models. The search for conserved domains revealed that, in contrast to Dm-Dcr-2, Sf-Dcr-2 retains the PAZ domain and unlike Tc-Dcr-2, it holds a carboxy terminal dsRNA binding motif (Figure 3(i)). The clustering array in phylogenetic study reveals that it shares more similarities with Bm-Dcr-2 in terms of domain conservation (Figure 3B).

Another variant of RNaseIII endonuclease known as Drosha is essential for miRNA biogenesis. Drosha along with its cofactor crops out ~70 nucleotide pri-miRNA product from the precursor pri-miRNA [41–43]. Two tandem RNaseIII domains of Drosha consecutively cleaves the 3′ strand and 5′ strand of double stranded RNA segment of pri-mRNA, generating a 2- nucleotide (nt) 3′ overhang. *Sf-Drosha* was annotated from *S. frugiperda* assembled genome based on the presence of a pair of RNA-specific endonuclease III domain and a dsRBM domain similar to *Bombyx mori* like protein (Figure 3A(ii)). To assess the involvement of Drosha in *Sf21* RNAi pathway, we performed the siRNA screening against Drosha in *gfp* based reversion assay.

**Figure 4:**
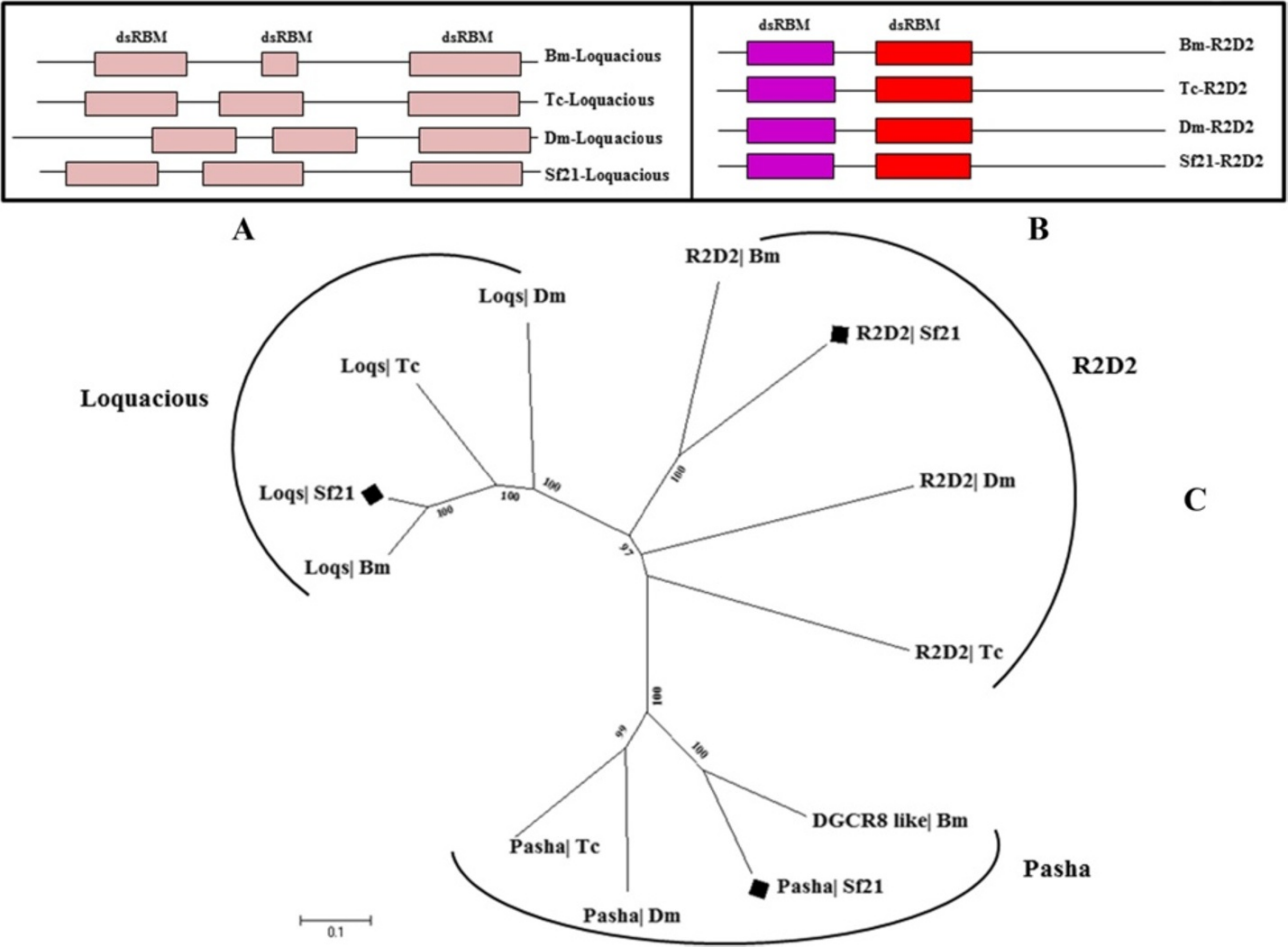
**Domain analysis and phylogenetic tree of dsRBM effector proteins. (A)** Tc, Bm and *Sf21* has three tandem dsRBM in Loquacious. **(B)** The domain architecture of R2D2 is much conserved while all referring insects have two RNA binding domains. **(C)** Phylogenecic analysis revealed the dsRBM effector components of *Sf21* Pasha, R2D2 and Loquacious are closest with respective *Bombyx* homologs.

Every Dicer or Dicer-like protein is assisted in its function by its cognate double-stranded RNA-binding domain proteins [12, 44, 45]. For example, Drosha functions in association with Pasha, human Drosha works along with DGCR8; Arabidopsis DCL1 is assisted by HYL1 protein etc [46]. Accordingly, we expected that, along with Sf21-Dcr-2, we should be able to locate Dm-R2D2 type of proteins. We found orthologs of *Bombyx* R2D2 in the *Sf21* genome (Figure 4B). Interestingly, a Loquacious homolog in *S. frugiperda*, which contains three domains of double-stranded RNA binding motif superfamily, was identified too (Figure 4A). The Sf-Loq dsRBM is highly analogous with Bm-loq in comparison to *Tribolium* or *Drosophila* homologs (Figure 4C). Dcr-1 mediates miRNA biogenesis involving functional pre-miRNA processing activity in the presence of its cognate partner Loquacious. Three RNA binding motifs of Sf-Loquacious may provide a strong interaction between Dcr-1 and dsRNA. Taken together, these findings suggested that *Sf21*, which have the same number of Dicer as well as Dicer associated proteins with *Drosophila* and *Tribolium*, with similar domain conformation and phylogenetic proximity with *Bombyx mori* homologs, might be involved in both RNAi and miRNA pathways (Figures 3 and 4), persisting with their conservation of dsRNA processing components in the passage of evolution.

Analysis of available transcriptome data set revealed an additional dsRBM which aligns with Pasha/DGCR8 homolog. Correlative phylogenetic study with domain frame characterizes high resemblance of Bm-DGCR8-like protein with Sf-dsRBM, implying it might be Pasha-like protein for *Spodoptera*. Hence it is tempting to speculate that the pair of Sf-Drosha and Sf-Pasha is present to process the miRNA biogenesis in the standard manner.

#### Argonaute proteins

**Figure 5:**
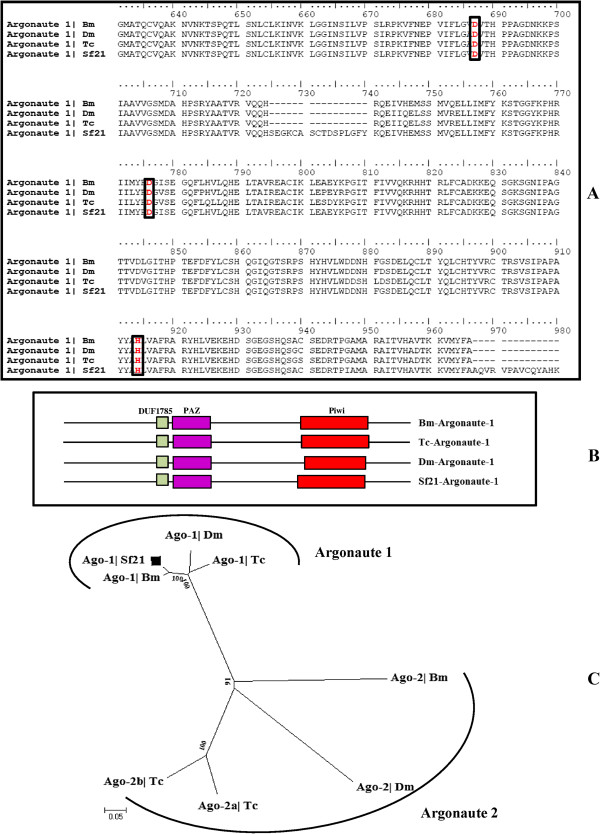
**Alignment Domain analysis and phylogenetic tree of Argonaute family of proteins. (A)** Amino acid alignment of Argonaute-1 protein of *Bombyx mori*, *Tribolium castaneum*, *Drosophila melanogaster* and *Sf21*. Indicated sequences were aligned using CLUSTALX. The conserved amino acid residues Asp, Asp and His (DDH) triad residues are marked with red. **(B)** Domain architecture of Argonaute proteins. The similarity in the domain architectures of Bm-Ago-1 and Sf21-Ago-1 suggests that they are orthologous and the organisation of PAZ/Piwi domain is highly conserved within the insects. **(C)** The phylogenetic tree was based on consideration of two different class of Argonaute proteins, miRNA class (Argonaute-1) and siRNA class (Argonaute-2). *Sf21* genome contains Ago-1 while *Tribolium*, *Bombyx* and *Drosophila* have both Ago-1 and Ago-2.

The Argonaute proteins are RNaseH family of proteins and are key components of RISC or miRNP complexes that are involved in post-transcriptional silencing, causing either mRNA-cleavage or transcriptional silencing [47]. Argonaute proteins are characterized by PAZ and Piwi domains; PAZ domain is involved in siRNA binding, while the Piwi domain possesses the RNase activity [15, 48] (Figure 5B). Number of Argonaute proteins varies in different organisms: Eight Argonaute genes exist in humans (4 Piwi subfamily and 4 eIF2C/AGO subfamily) [49, 50]. *Tribolium* and *Drosophila* have five members, while brown plant hopper *Nilaparvata lugens* has two orthologs of *Drosophila* Argonaute genes [27, 29, 51–53].

**Figure 6:**
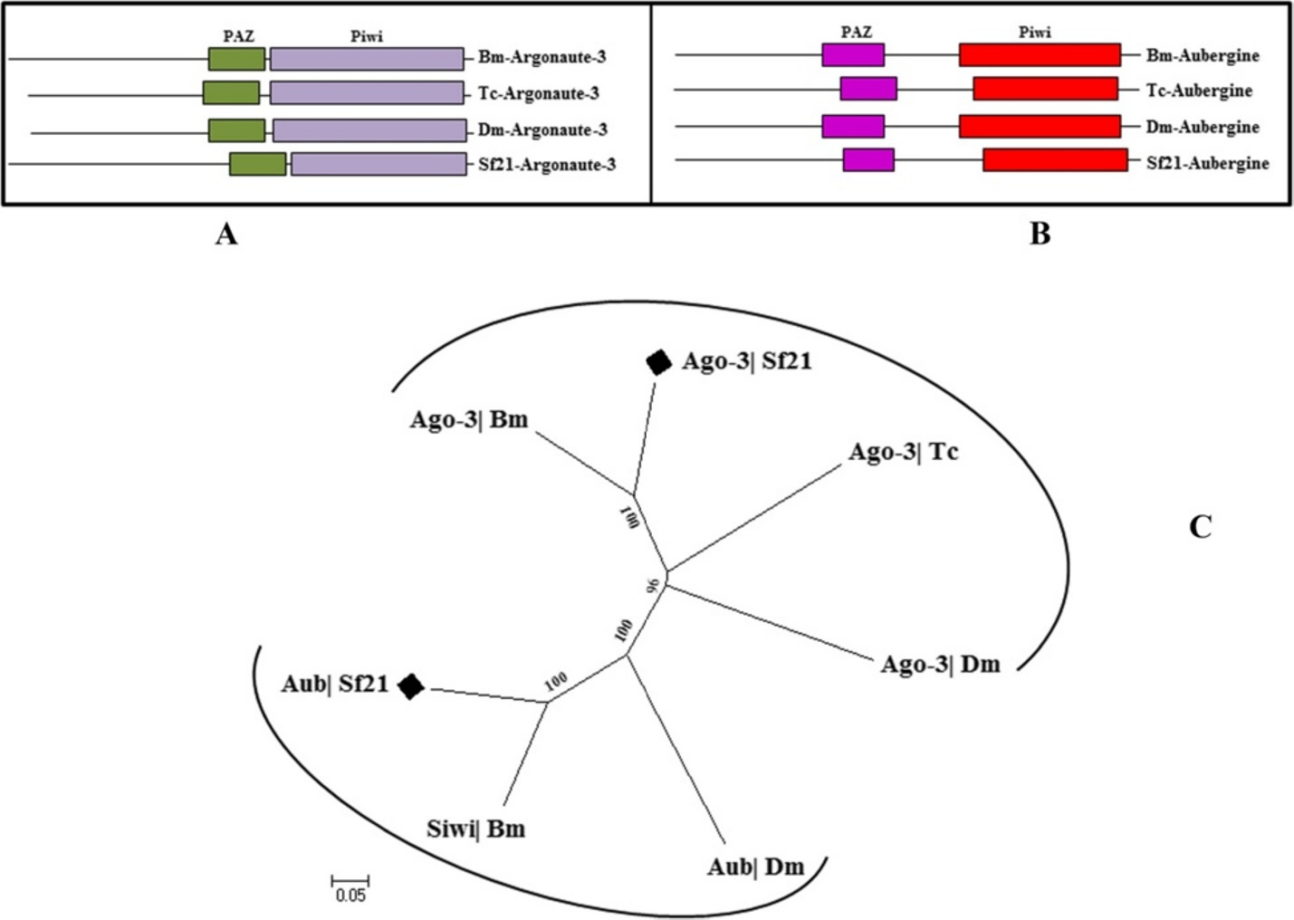
**Domain structure and phylogeny of piRNA class components. (A)** and **(B)** domain search finds both *Sf21* Argonaute-3 and Aubergine like protein have conserved lineage of PAZ and Piwi domains. **(C)** A phylogenetic tree was created using the full-length sequences of piRNA class proteins. Siwi represents silkmoth Piwi.

We identified Ago-1 and Ago-3 from the annotated genome and assembled transcriptome of *S. frugiperda* (Figures 5B and 6A). The amino acid sequence alignment of Piwi domain in *Sf21* revealed that the catalytic motif (aspartate, aspartate and histidine; DDH) is well conserved with other insect species like silkworm, fly and beetle Argonaute members (Figure 5A). However, transient siRNA knockdown assay monitoring *gfp* expression validated the role of Sf-Ago-1 as potent miRNA class Argonaute. In view of evolutionary lineage and considering different orthologs of Argonaute proteins, *S. frugiperda* Argonaute was classified as Ago-1 subclass highly analogous to Bm-Ago-1 (Figure 5C). Subsequent comparison of consensus sequences with homologous proteins shows that Sf-Ago-1 appears in the Ago subfamily, whereas Sf-Ago-3 appears to be clustered in the Piwi subfamily (Figure 6C). In view of structural similarity with *Bombyx mori* Ago-3 component, we assume functionally, Sf-Ago-3 with its sequence of signature PAZ and Piwi domains might be a possible partner for its piRNA-like small RNAs [54].

#### Aubergine

Piwi (P element-induced wimpy testes) are germ line specific Argonaute family proteins that associate with piRNAs and promote cleavage of expressed transposon RNA targets leading to silencing of transposition [55–57]. Two Piwi subclass of RNA interference (RNAi) accessory proteins; Aubergine and Piwi were identified from *Drosophila* ovaries [52, 58] and genome wide survey of *Tribolium* also identified a Piwi/Aub class Argonaute involved in transcriptional silencing along with Tc-Ago-3 [29]. We could identify a single Aubergine protein homolog related to the Piwi/Aubergine subfamily proteins in *Sf21* cells (Table 1) (Figure 6B, 6C). As *Sf21* cells are developed from ovaries of pupal stage *Spodoptera frugiperda*, it might possess an active piRNA biogenesis pathway and Sf-Aubergine could induce Piwi-associated RNA silencing that is reminiscent of *Drosophila* and *Bombyx mori.*

### *Spodoptera frugiperda*auxilliary RNAi factors

#### ‘DEAD’ box containing RNA helicase domain proteins

DExD/H-box RNA helicase family of enzymes functions as RNA chaperons because of the presence of a unique RNA recognition motifs over an ATP dependent component [59, 60]. Conserved DExH-box helicase, predominantly Dicer and Ago-related helicase genes have been shown to be required for RNAi in *C. elegans* and human [61, 62]. ATP-dependent subunits p68 and p72 of DEAD box helicase distinguishes pri-miRNAs and induces their processing through Drosha complex in microRNA biogenesis pathway [63].

**Figure 7:**
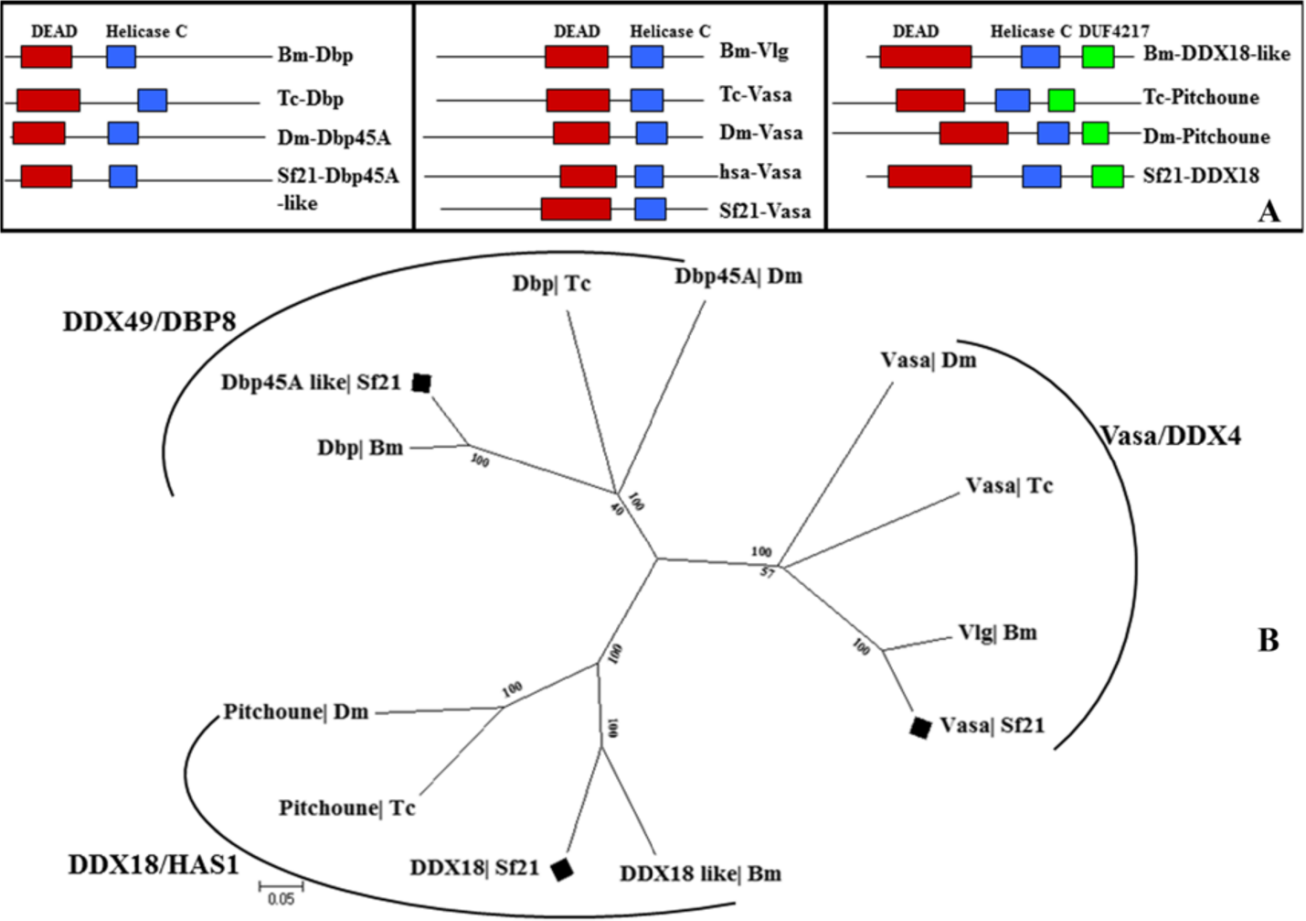
**Domain analysis and phylogenetic tree of DEAD RNA helicase domain proteins. (A)** Conserved Domains Database search shows both Dbp45A and Vasa RNA helicases has DEAD domain and a C-terminal Helicase domain while DDX18/Has1 subfamily of helicase has an unknown domain which is present in all concerned helicase proteins of this family indicating clear orthology. **(B)** The neighbour-joining tree is based on the alignment of the conserved DEAD domain containing RNA helicases. The DEAD helicases forms three distinct clusters categorized into three subfamilies Dbp45A, Vasa and DDX18/Has1. Sf-DDX18 like proteins is closest with *Bombyx mori* but similar with Pitchoune of *Drosophila* and *Tribolium* too. Vasa subgroup family of proteins being important for a piRNA pathway suggests Sf-Vasa might participate in Piwi associated RNA silencing.

We identified three distinct classes of DEAD box RNA helicases related to siRNA pathway in *Sf21* genome. One of the probable ATP-dependent RNA helicase resembles Dpb45A (DDX49/DBP8 subfamily) of *Drosophila melanogaster* and DDX49 of *Homo sapiens* that also has a close homology with another DEAD box helicase family protein of *Bombyx mori* in turn (Figure 7A, 7B). Another member from the DEAD helicase that belongs to Vasa/DDX4 RNA binding subfamily was identified. Vasa RNA helicases are known to recruit Aubergine which implicates transposable element silencing in *Drosophila*[64]. A third Helicase from the set of putative RNAi factors was found to be analogous with RNA helicase Pitchoune of *Drosophil*a and *Tribolium* and DDX18 of *Bombyx mori* and *Homo sapiens,* which belongs to DDX18/HAS1 DEAD box helicase subfamily. Presence of a C-terminal DUF4217 (domain of unknown function) besides the ATP-binding and Helicase domains confers a typical feature, which characterizes Sf-DDX18-like protein under DDX18/HAS1 subfamily (Figure 7). Of the three RNAi related helicases identified in *Sf21* genome, knocking down the DDX18/HAS1 mRNA provided the highest reversion level of *gfp*, thereby.

#### RNA processing module

A number of candidate RNAi genes encode proteins with known or predicted features in RNA binding or processing. Using the reversion of RNAi sensor assay, we identified five factors that participate in RNAi process and possess RNA binding as well as processing modules (Table 1); U1 small nuclear ribonucleoprotein (U1snRNP), SmG, smg-2 like, Integrator complex subunit (Int11) and Zn finger protein. U1 small nuclear ribonucleoprotein (U1snRNP) is known to inhibit the gene expression by preventing poly(A) polymerase (PAP) mediated 3′ polyadenylation. This factor thus couples the splicing and silencing machineries [65, 66]. In the reversion assay, *Sf21* U1snRNP showed a moderate level of reversion of RNAi, thus confirming its role in RNAi in *Sf21* cell line.

Small nuclear ribonucleoprotein G (SmG) of both *Drosophila* and *Bombyx* have been implicated to participate in RNA binding and spliceosomal machinery [67] but not yet analysed as RNAi factor. However, *C. elegans* SmG protein actively coordinates nematode RNAi [20]. Gene predictions from *Spodoptera frugiperda* genome assemblies found SmG with a distinct LSM domain. SiRNA guided knockdown assay of Sf-SmG suggested a conserved function of this protein in *Sf21* cells gene regulation.

Genome wide RNAi screen in *C. elegans* classified precursor proteins associated to post-transcriptional pre-mRNA processing like cleavage and polyadenylation associated factors that play roles in RNAi pathway [20]. The integrator complex pairs with carboxyl-terminal domain (CTD) of the largest subunit of RNA polymerase II for co-transcriptional 3′-end processing of U1 and U2 small nuclear RNAs (snRNAs) [68]. We identified a putative factor namely Integrator complex subunit with one domain of Lactamase B superfamily and a C terminal beta-CASP domain in *Sf21* genome. Comparative genomic analysis showed that Sf-Int11 is orthologous to the integrator complex subunit 11 of *Bombyx mori*. Surprisingly, the functional assay for Sf-Int11 showed a very wide range of *gfp* reversion in *Sf21* cell line, therefore requires further investigation for Int11 involvement in siRNA pathway.

An array of Zn finger proteins has been an integral effector of both *C. elegans* RNA-silencing pathway and miRNA pathway [20, 69]. Lately a chromatin associated RNA processor zinc finger protein has also been identified as a potent component in Piwi-interacting RNA driven transcriptional silencing paradigm in *Drosophila*[70, 71]. We identified PHD superfamily Sf-Zn finger protein homologous to Dm-INTS12 with defined PHD-finger [72].

Nonsense-mediated decay (NMD) associated proteins like Smg are essential for RNAi mediated mRNA degradation [73]. Studies suggest that series of phosphorylation and de-phosphorylation of NMD factors of mRNA surveillance complex initiates sequestration of nonsense messenger RNA to P bodies [74]. We found one of the core-proteins of the NMD complex Regulator of nonsense transcripts-1 homolog i.e. UPF1 in yeast or smg-2 like protein of *C. elegans* in *Sf21* genome. Sf-Smg-2 has a conserved N-terminal RNA helicase UPF2 interacting domain. However, we observed low levels of *gfp* reversion with the three siRNAs used in the present study (Table 1).

#### Protein Kinase family of signalling and allied components

Many components of RNAi machinery have been shown to undergo phosphorylation and are regulated by EGFR/MAPK signalling pathway [19, 75–77]. The genome wide search for the RNAi-genes predicted a number of kinase family of enzymes in *Sf21* cell line that have distinct roles in signal transduction pathways. The comparative genome analysis for *S. frugiperda* RNAi components followed by functional RNAi reversion assay identified eight signalling related components; calcium/calmodulin-dependent protein kinase I (CaM Kinase), two protein kinases (PKC), Serine/threonine p21-activated kinase (PAK) mbt like protein, an inhibitor of nuclear factor kappa-B kinase subunit beta (IKK-beta), MAP Kinase Kinase 4 and STE20/Fray protein kinase (Table 1).

#### Multi drug resistance cassette transporter

Mechanistic details and functional analysis identified about ten ATP-binding Cassette (ABC) Transporter genes participating in active RNAi function in *C. elegans*[17]. A half-transporter member HAF-6 of ABC_RNAi_ subclass and its interacting partners MUT-7/RDE-2 have been shown to induce RNAi [78, 79]. Although ATP-binding cassette (ABC) transporter superfamily genes have been widely annotated in *Tribolium* and *Bombyx*[80, 81], their potential roles in mounting RNAi response in insects have not been reported. We identified a multi-drug resistance protein 1A-like (Sf-MDR1A) of *S. frugiperda* with two conserved ABC transporter transmembrane region that produces a robust response in the cell based RNAi-reversion assay (Table 1).

#### Tudor

Tudor-SN, a staphylococcal/micrococcal nuclease, which harbours a Tudor domain, is a canonical component of active RISC complex that potentiates dsRNA-mediated transcriptional silencing in *Caenorhabditis elegans*, *Drosophila* and mammals [16]. A number of proteins with Tudor domain interact with components of gene silencing machinery like Piwi, Armitage, Yb and Zucchini in *Drosophila* somatic and germ line piRNA pathway [82]. Recent studies in *C. elegans* ERI endo-RNAi shows that tandem Tudor domain protein ERI-5 stably loads RNA-dependent RNA polymerase (RdRP) complexes to DCR-1-dependent and DCR-1-independent pathways [83]. Surprisingly, RNAi efficiency in *Bombyx mori* BmN4 cells remains unchanged despite the knockdown of Bm-Tudor-SN [84]. We identified a *Sf21*-Tudor domain containing protein with two tandem staphylococcal/micrococcal nuclease and one Tudor domain. Importantly, knockdown of *Spodoptera* Tudor did not affect RNAi-reversion efficiency in our cell based assay (Table 1).

#### Sid-1 like protein

**Figure 8:**
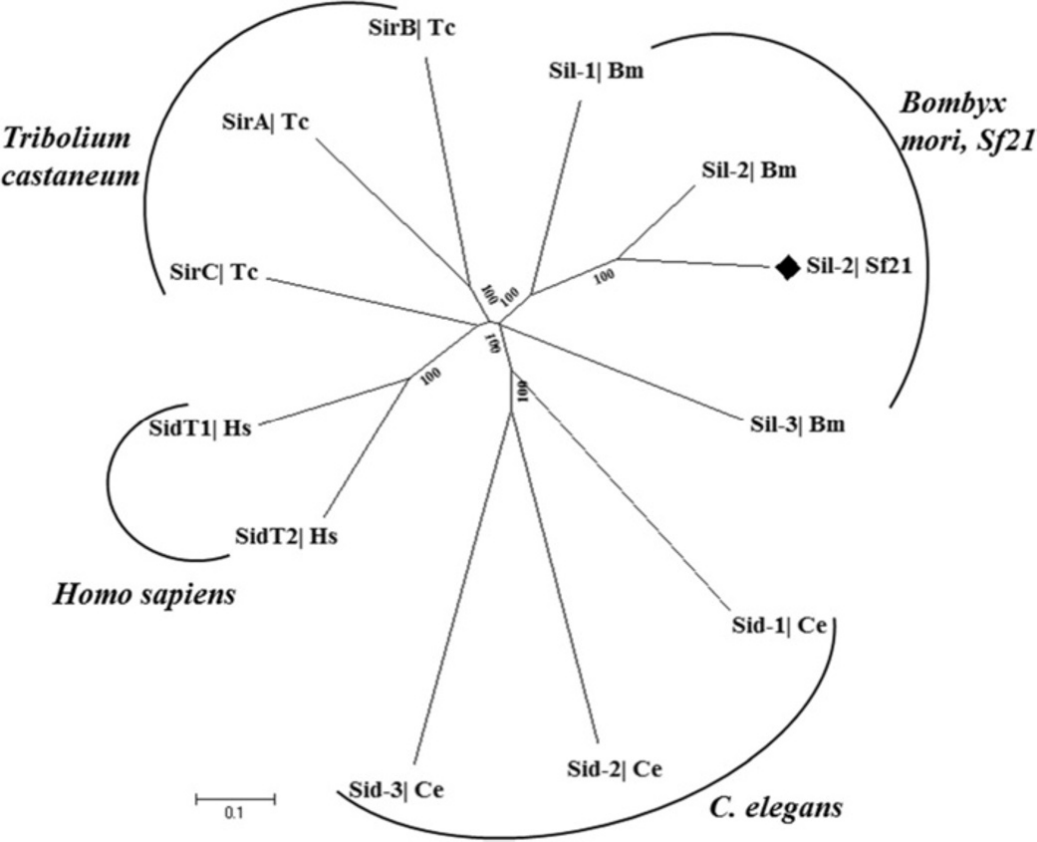
**Sil protein phylogenetic analysis.** The neighbour-joining tree is based on the alignment of *sil* gene family of proteins in different organisms. Sf-Sil-2 sub-clusters with the silkmoth Bm-Sil-2, while Tc-SirA and Tc-SirB compose a distinct cluster. Orthology of these insect is clear from this phylogenetic analysis that Lepidoptera Sid like proteins are evolutionary conserved. Ce-Sid1-3 and Hs-SidT1 and SidT2 diverge out separately and Tc-SirC joins Hs cluster.

In *C. elegans*, systemic RNAi involves Sid-1 mediated intracellular transport of dsRNA, while in human cells, siRNA internalization requires Sid-1 gated transportation [18, 85]. Among the insect species, *Drosophila*, the premier model system of RNAi research lacks systemic RNAi and Sid-1 as well [86], while *Tribolium* possess three sets of Sid-1-related genes (Tc-sirA-C) [29] and also, Sid-1 promotes RNAi effect in BmN4 cell line of silkmoth with regards to dsRNA uptake [87]. We identified Sid-1-like channel protein gene homolog Sf-Sil in *Sf21* genome and *gfp* reversion analysis revealed that Sf-Sil might be a putative RNAi effector component. Phylogenetic profile of vertebrate Sid gene family showed separate clades for Ce-Sid, Bm-Sil, Hs-SidT or Tc-Sir. It appears that *S. frugiperda* Sil belong to insect clades forming a distinct sub-cluster within Bm-Sil branch depicting a significant convergent sequence similarity with the silk-moth as Sf-Sil-2 protein (Figure 8).

#### Chromatin factors

Recent studies indicate that heterochromatin is not completely inert; it is also transcribed, and rapidly silenced, by a sequence of events directed by RNA interference components [88]. In addition, heterochromatic modifications are also crucial for epigenetic regulation: gene and transposon silencing, X chromosome inactivation (XCI) and imprinting [89–91]. RNAi regulates the assembly and spreading of heterochromatin at the peri-centromeric *dg* and *dh* repeats in *Schizosaccharomyces pombe*[92]*.* Genome-wide RNAi screens have shown that chromatin associated proteins, histone deacetylase complex (HDAC), and methyltransferases are essential for *C. elegans* RNAi [20]. We identified Sf-Histone-3 Lysine-4 N-methyltransferase with conserved PHD-like zinc-binding domain and Sf-Histone deacetylase-3-like protein in genome-wide *in-silico* RNAi screen of *Spodoptera* genome and these proteins showed moderate levels of reversion in the RNAi reversion assay, suggesting their participatory role in RNAi (Table 1).

Besides methyltransferase, cognate histone acetyltransferase (HAT) component Gas41, which ushers in transcriptional activation [93], have been found to play a secondary role in canonical RNAi pathway [94]. Gas41 thus provides a link between nuclear structure and gene silencing in *C. elegans* RNAi. Though RNAi sensitivity of transcription factors has not been reported in insect system, we found Sf-Gas41 in our RNAi screens. Sf-Gas41 has YEATS family domain resembling *Drosophila* counterpart, thus reflecting conservation within insects for the RNAi machinery.

#### Translational unit

Homology based functional analysis and evolutionary conservation showed that Argonaute family of proteins are probably the primitive extension of components of translation initiation apparatus [95]. Genome scale RNAi screening recognized eIF2B and 60S ribosomal subunit L23a as the RNAi pathway candidates, along with eIF3D as miRNA pathway candidate gene in case of *C. elegans*[20, 69]. A recent study exhibited that eIF4AII activity is critical for miRNA mediated gene silencing by restraining translation initiation via recruitment into miRISC [96, 97].We identified Sf-eIF2B-gamma, DEAD-box ATPase and ATP-dependent RNA helicase eIF4A paralog proteins Sf-eIF4AII and Sf-eIF4AIII in our screens. In addition, Sf-RPL23P a Ribosomal L23 super family of protein was recognized as siRNA-guided silencing factor. Identification of these proteins raises the intriguing prospect of a link between RNAi pathway and translational components.

#### Cell division associated elements

In different model organisms, it has been demonstrated that RNA mediated gene regulatory pathway is a modular scaffold, which can coordinate with fundamental units of cell cycle to attune cellular physiology [88]. In *Drosophila*, functional activity of Ago-1 requires cytokinesis regulator sticky/citron kinase protein for silencing at transcriptional level [98] whereas in *S. pombe,* Ago-1 interacts with 14-3-3 proteins and this interaction is required for functioning of Argonaute protein in cell cycle and/or gene-silencing pathways [99, 100]. Further, Stoica *et al*. [100] demonstrated the microtubule walker Kinesin Motor Protein Cut7 as an interacting partner of Ago-1-RISC component [101]. In the present study, we identified two Kinesin like proteins; KIF18A-like and KIF3A-like along with a Cyclin-dependent kinase 5 homolog in *Sf* RNAi screen. Among these proteins, Kinesin like motor proteins has been first identified in insect to participate in RNAi machinery. However, further experiments will be required to identify their functional role.

#### Metabolic factor

Little is known about the metabolic processes that drive the RNA silencing process. In *C. elegans,* investigators identified mitochondrial isocitrate dehydrogenase as a metabolic factor that participates as a nematode RNAi candidate [20]. We also identified Sf-isocitrate dehydrogenase [NAD] subunit alpha as an important factor in RNAi screen using *gfp* expressing *Sf21* cell line. As shown in Table 1, significant reversion was observed when siRNA corresponding to *Sf21* isocitrate dehydrogenase gene were used.

#### Miscellaneous RNAi factors

Based on robust expression in the *gfp* reporter system, we were able to detect few other RNAi related proteins that did not cluster with previously known functional RNAi factors. These include Myosin VIIa-like protein, Nucleolar complex protein 2 homolog, WD 40-like repeat domain and S-phase kinase-associated protein (SkpA). Rik1 the crucial element in heterochromatic RNA silencing in fission yeast has C-terminal WD-repeat domain [102]. Another screen for *C. elegans* microRNA candidate identified ubiquitin-dependent catabolic protein Skp-1 homolog responsible for protein turnover during RNA silencing [69]. It will be interesting to functionally characterize the role(s) of these proteins in RNAi pathway in the *Sf21* line.

## Discussion

With of the advent of NGS technologies, the presence of a host of small RNAs of diverse types along with their relative abundance has been highlighted across various eukaryotic organisms. The biogenesis and functions of these small RNAs are important research aspects as these RNAs control a gamut of biological functions. A vast majority of these pathways are conserved but also diverse not only across various eukaryotic species but also within tissues of organism. In recent years, such diversities have been reported from a variety of organisms including ciliates, fungi, nematodes, plants and animals. These pathways have also highlighted the diversities in the factors that are responsible for RNAi competence and their interacting partners [103–107]. Thus it is not surprising to investigate the varied nature and abundance of the RNAi factors in different organisms. For example, genome-wide approach has identified ~90 proteins involved in RNAi in *C. elegans*, while in *Drosophila melanogaster* only ~20 components have been shown to be associated with RNAi process.

There has been a recent splurge in the research pertaining to insect genomics. This is significant not only from crop protection and food security points of view but also from the angle of application of comparative genomics for discovery of novel insecticidal molecule [108]. Among various insect species, genome-wide approaches have been applied on *Tribolium castaneum* and *Nilaparvata lugens* and seventeen to fourteen RNAi associated genes have been described respectively. RNAi is being used in our laboratory to carry-out functional genomic studies in *Sf21* cell line as well as in intact insects; *Spodoptera litura* and *Helicoverpa armigera*[109, 110]. We have recently assembled *Sf21* cell transcriptome and genome data. Using these data set and by setting up a functional screen for the putative RNAi factors, in the present study we explored the components that make the *Sf21* insect cells competent for the RNAi. To the best of our knowledge, this is the first genome wide approach for a Lepidopteran RNAi factors with *in sillico* identification and their validation.

To identify components of RNAi machinery in *Sf21* cell a homology search was carried out in *Sf21* assembled genome based on previously known RNAi factors from *C. elegans*, *B. mori*, *T. castaneum* and *D. melanogaster*. These searches lead to the identification of about eighty genes and many of these RNAi candidate homologs were close to *B. mori* gene inventories. The minimal branch divergence of *Sf* proteins from the cluster of *Bombyx mori* homologs in the appropriate phylogenetic tree certainly justifies the notion that the Lepidopteron insects evolved the well conserved functions in the evolution. The molecular phylogeny of the insects also highlights the tremendous degree of sequence similarities of the overall RNAi proteins across the insect kingdom.

To confirm the participation of *in silico* identified eighty *S. frugiperda* genes in RNAi pathways, a systematic knock down of each of these 80 genes was performed in reversion of silencing assay using *Sf21-gfp* reporter cell line earlier reported by Singh *et al.*[30]. Forty one (Table 1) of the eighty putative RNAi factors (Additional file 1) considerably showed block in RNAi in these cell lines, thereby indicating a role(s) of these factors in RNAi pathway. This number of RNAi factors is higher than any other insect species examined so far and it is close to the number (~90) of RNAi factors described for *C. elegans*[20]. Thus, our results together with available data of *C. elegans* provide a platform for appreciating diversity in RNAi composition across eukaryotes.

Among the major RNAi factors identified in *Sf21* are Dcr-1, Dcr-2, Drosha, Aubergine, Pasha, Ago-1, Ago-3, Loquacious and R2D2. Although a close similarity was observed between these main RNAi factors and the corresponding *Bombyx mori* factors, however subtle differences were also observed for the factors and such differences have been existent among related insect species. For example, only a single Dicer homolog, Bm-Dicer-2 has been identified in *Bombyx mori* genome while we identified two Dicers; Dcr-1 and-2 in *Sf21* genome.

It is interesting to note that our biological screen also revealed the Dicer gene, albeit in an unexpected manner. Here, we can only speculate about this surprising finding. As reported earlier, Dicer acts as an intergral siRNA-duplex-binding complex by recognising 2-nt 3′ overhangs. Dicer itself binds siRNAs that mimic the yields of Dicer cleavage (19 bp + 2-nt 3′ overhangs) and serve as a prime senor to harbour effective siRNA duplex which further leads to Argonaute loading [111]. Thereby, in our study, we propose the down regulation of dicer by incorporation of Dicer specific siRNA might interfere in the steps of RISC loading. Moreover, down regulation of Dicer might have some indirect effect on the abundance of other RNAi factors for maintenance of homeostatic equilibrium.

*Sf21* Drosha protein show similar domain organization as found in *B. mori*, *T. castaneum* and *D. melanogaster* homologs, however Tc-Drosha lacks a c-terminus dsRBM domain. We also identified other pre-Dicer component; Pasha, thereby suggesting a cross-talk between the miRNA and siRNA pathways in these *Sf21* cells. It has been proposed that siRNA and miRNA pathways are partially overlapping and few factors participate in both the pathways. In *Drosophila*, Dcr-2/R2D2 complex is involved in the processing of siRNA pathway from exogenous origin, while endogenous siRNA processing requires Dcr-2/loquacious complex [112]. Since we identified two Dicer genes; Dcr-1 and Dcr-2 in *Sf21* genome along with R2D2 and Loquacious, it is possible that Sf-Dcr-2/R2D2 complex is participating in siRNA dependent silencing mechanism and the complex of Sf-Dcr-1/loquacious could be involved in the miRNA pathway of *S. frugiperda*. It is also possible that there is synergy between these two pathways. Emerging evidence indicates that both *Drosophila* and *Tribolium* requires R2D2 for RNAi initiation [13, 29]. Surprisingly, the Silk moth Bm5 cell line is devoid of R2D2 expression [26]. In *Sf21*, we identified the dsRNA-binding domains of R2D2, which in association with Dcr-2 might be critical for siRNA binding and triggers the assembly of si-RISC complexes.

Among the prime catalytic RNA-endonucleases, Argonaute-1 and Argonaute-3 were identified in *Sf21* genome, whereas *Drosophila*, *Tribolium*, *Bombyx* and *Nilaparvata* have all three different classes of Argonauts; Ago-1, Ago-2 & Ago-3 proteins. It is possible that Sf-Ago-1 is required for siRNA routed RNAi activity in *Spodoptera*, which is coherent with the report that Dm-Ago-1 functions downstream of siRNA generation [113]. Taking in view the finding of a recent study, we performed amino acid alignment of Sf-Ago-1 that identifies MC motif in the MID domain with two conserved phenylalanine residues at F522 and F557 like Bm-Ago-1. Such correlation establishes the fact that besides translation repression, Sf-Ago-1 being homologous to Bm-Ago-1 might influence P-body localization [114].

Along with the classical RNAi pathway components, we also identified few bona fide piRNA pathway components such as Ago-3, Aubergine, Vasa RNA helicase and Tudor domain protein. Silkworm Piwi (SIWI) and Bm-Ago-3 endogenous expression in ovary-derived BmN4 cell line has been exclusively found during pupation to execute both primary and secondary steps of piRNA biogenesis [54, 115]. In addition to Piwi/Aubergine, Vasa helicase and Tudor proteins have been reported for canonical ping-pong cycle in *Drosophila* ovary. Since *Sf21* cell line have been developed from pupal ovarian origin and from the outcomes of well-known piRNA pathway tissue-specific factors in high throughput assay, we propose an interface between the classical piRNA and siRNA pathways in *Spodoptera*. Phylogenetic and protein domain analyses of these factors also indicate a convergent similarity with silkmoth piRNA unit. Recently, transgenic RNAi screen in ovarian germ cells led to the discovery of 74 piRNA biogenesis factors in *Drosophila*[116]. Therefore, a further functional analysis might unravel the involvement of genes like Ago-3, Aubergine, Armitage or Spindle-E for *Sf* piRNA gene family.

Based on the results of functional RNAi assay, a number of accessory RNAi factors were discovered in the *Sf21* genome. These were classified into eleven groups based on their functional domains and reported functions. Similar classification has also been done for *C. elegans* RNAi factors [20]. Prominent among the factors, which showed maximum RNAi inhibition, are the members of DEAD-box family of RNA helicases, ABC transporter family, factor(s) possessing RNA binding module such as Integrator complex subunit (Int11) and a metabolic factor, isocitrate dehydrogenase. Few of these factors have been reported to interact with the main components of RNAi machinery. For example, conserved dicer-related DExH-box helicase genes *drh-1* and *drh-2* interact with RDE-4 protein which is required for direct RNAi in *C. elegans*[62]. In human, DEAH family of RNA helicase named RNA helicase A (RHA) interacts with Ago-2, Dicer and TRBP through its dsRBD. RHA acts as an activation component of siRISC with a functional role in RNAi-mediated silencing [61]. Recent study in HeLa S3 cells indicates DEAD-box helicase 3 (DDX3) as potent factor with sub-cellular localization coupled to Ago-2 in RNAi pathway [117]. Surprisingly, the *Sf*-ABC transporter family, present in *C. elegans,* is a lonesome candidate as it is also found only in *Sf21* amongst the insects so far [17, 78, 79]. Hence, our data indicate that RNA interference in *Sf* cells might require ABC transporter dependent trafficking mechanism. It is quite significant that, in spite of multiple duplication in the lineage from nematodes to arthropods, ABC membrane transporters are non-redundantly required for RNAi function.

The ability of dsRNA import and induction of systemic RNAi is been highly conserved in *C. elegans* and has been correlated with the expression of *sid-1* gene. *Tribolium* and *Nilaparvata lugens*, both of the insects in which systemic RNA interference is prominent, have Sid-1 like proteins which have higher sequence similarity with insect Sil than with that of *C. elegans Tag-130*[27, 29]. However, absence of Sid-1 homolog in *Drosophila* correlates with lack of systemic spreading of RNAi response in fly. The aphids are RNAi-competent but there are controversies about the systemic response in aphids despite the presence of *Sid-1* homologs [118]. The *Sid-1* gene has been found in highly diverse insect lineage such as Orthoptera, Phthiraptera, Hemiptera, Coleoptera and Hymenoptera but is absent in Diptera [119]. As far as *Spodoptera frugiperda* is concerned, there are both structural and functional evidence in favour of systemic RNAi-response. We have previously shown the spreading and retention of silencing at the midgut of the closest insect *Spodoptera litura* causing systemic RNAi [109]. In the present study, we confirm the existence of *Sf-sil-2* gene. This coupled with the observation of RNAi transmission in related organism *S. litura*, suggests the presence of systemic RNAi in *Spodoptera frugiperda*. As our functional assay was cell-line based, the presence of *Sf-Sil-2* gene also leads us to speculate the intra-cellular amplification of the siRNAs.

A distinct number of protein kinase family members are found to be associated with *Sf21* RNAi process, raising an interest for a provisional link between RNAi pathway and signalling mechanism. Both of these two processes might have dynamic & overlapping functions within a cell. A number of reports have shown the role of phosphorylation in Ago protein turn over [19] and Ago-2/RISC localization into distinct centres called cytoplasmic bodies or mammalian P-bodies, guiding efficient cleavage and degradation for siRNA-mediated mRNA decay or translational repression [120, 121]. Although more functional studies are required in this regards, we predict similar role(s) for these kinases in *Sf21* RNAi processes. Besides these, phosphorylation of small RNAs might also be required for efficient RNAi activities and a subset of these kinases might be involved in such phosphorylation.

**Figure 9:**
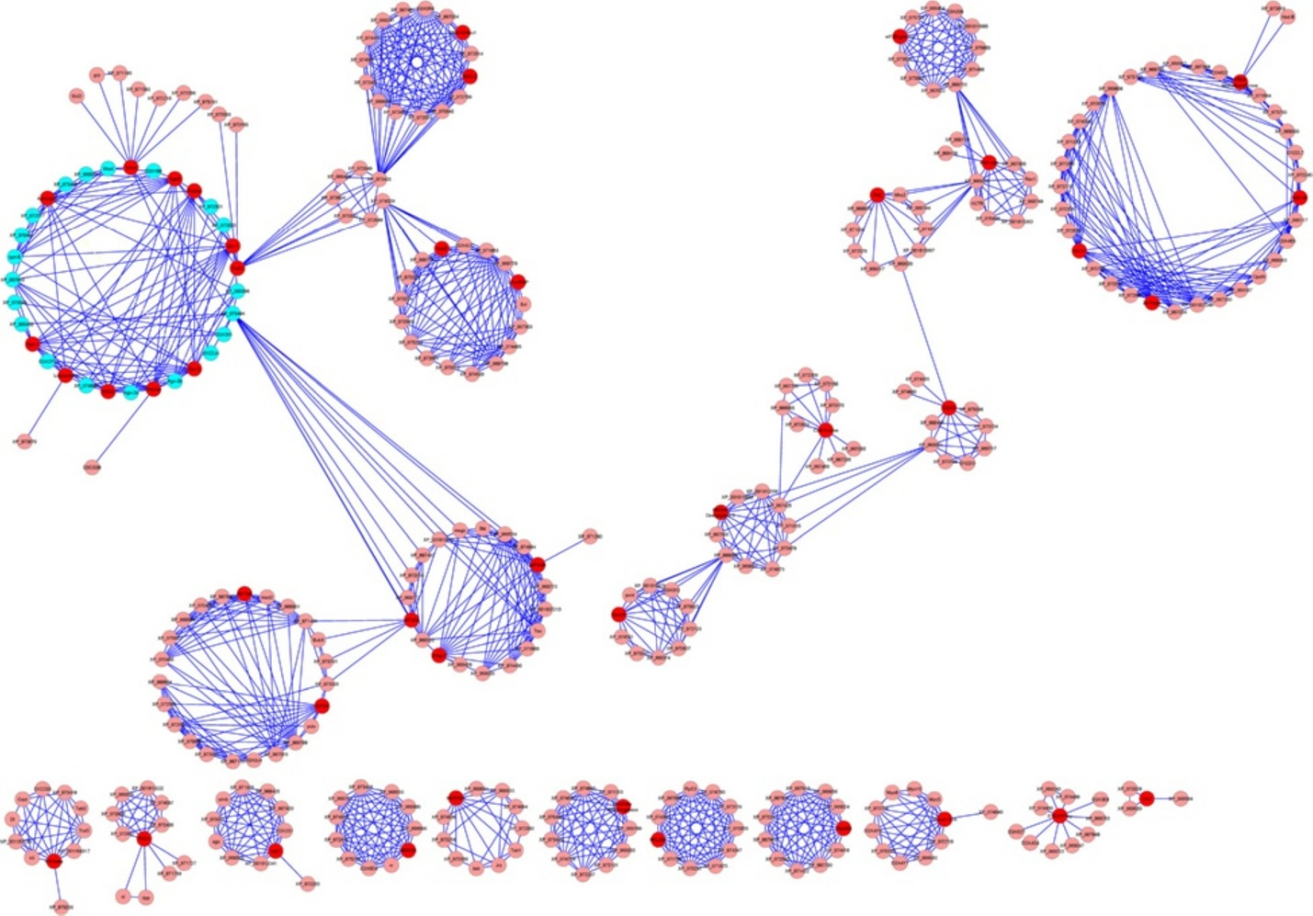
**Interactome Map of** ***Sf21*** **RNAi factors.** Protein-protein interaction data for 42 functionally validated RNAi factors identified in the siRNA mediated high through-put screening were constructed by homology based search in *Tribolium*. The protein interaction data shows 345 nodes with 1257 edges. The nodes in blue highlighted cluster identifies core RNAi components. The red nodes indicate *Sf21* RNAi factor homologous to corresponding *Tribolium* protein. The pink node identifies the interactors of putative candidates postulated from *Tribolium* network.

The availability of known and predicted protein interactions of closely related insect species like *Tribolium* helped us to construct a protein-protein interaction map to facilitate a molecular network consisting both core & auxiliary *Sf21* RNAi candidates (Figure 9). A search of interolog prediction of *Sf21* consensus sequences present in *Tribolium* included the above mentioned forty two Sf-RNAi factors. The map provides three distinct clusters with ten small networks of RNAi interactors. The cluster comprising core RNAi candidates postulates connections among new auxiliary factors which includes DEAD helicase DDX18/Has1, three of the RNA processing factors SmG, U1AsnRNP, Regulators of nonsense transcript 1 homolog (smg-2 like protein), Translation component eIF4AII, eIF4AIII, cell division elements KIF18A and KIF3A like proteins and Nucleolar complex protein 2 homolog. Another cluster links the other translational unit eIF2B-gamma, Signalling component PAK mbt like protein, PKC, CaM kinase, Chromatin factor Histone deacetylase 3, Gas41 and CDK5 as well. Integrator complex subunit (Int 11), Histone3 Lysin4 N-methyltransferase, WD 40 repeat domain and Zn finger proteins club to a separate group too. However, the interrelatedness between these two separate clusters and the same with core RNAi group are matters of further investigation. The clustering of other small individual networks like Sil-2, isocytrate dehydrogenase, Multidrug resistance MRD1A proteins also challenges the identification of provisional link of the RNAi mediated processes of *Sf21* cells.

*Spodoptera frugiperda* derived cell line *Sf21* is one of the most common insect cell lines used for expression of proteins via baculovirus expression system. In *Sf21* cells RNAi technology successfully allows loss of function phenotypes with stable expression. Understanding the participation of RNAi components might be useful information in *S. frugiperda* cells for more effective and persistent means of transgenic studies. Involvement of such a huge number of accessory elements in small interfering RNA networking gives a global view about organism specific repertoire of RNAi machinery (Table 2). This justifies the rationale where auxiliary RNAi factors are also integral part of gene silencing apparatus. It is possible to target these secondary elements to somehow regulate the strength of the silencing process. Hence this report will help expand the domain RNAi technology as a larvicidal tool and advance the frontiers of RNA interference biology of the major quarantine pests.

**Table 2:** **Comparative analysis of** ***Sf21*** **RNAi genes**

Gene ( ***Spodoptera frugiperda*** ) (Lepidoptera)	***Bombyx mori*** (Lepidoptera)		***Tribolium castaneum*** (Coleoptera)		***Drosophila melanogaster*** (Diptera)		***Nilaparvata lugens*** (Hemiptera)		***Caenorhabditis elegans*** (Nematoda)	
	**A**	**B**	**A**	**B**	**A**	**B**	**A**	**B**	**A**	**B**
Argonaute-1	+	-	+	+	+	+	+	+	+	+
Argonaute-3	+	+	+	+	+	+	+	+	Homolog *prg-1*	+
Dicer-2	+	-	+	+	+	+	+	+	-	-
Dicer-1	-	-	+	+	+	+	+	+	+	+
Aubergine	+	-	+	-	+	+	+	+	Homolog *prg-1*	+
Drosha	-	-	+	+	+	+	+	_+_	+	
Pasha	+	-	+	+	+	+	+	+	+	+
Loquacious	+	-	+	+	+	+	+	+	-	-
R2D2	+	-	+	+	+	+	+	+		
Dbp45A subfamily	+	-	-	-	+	-	-	-	-	-
VASA subfamily	+	+	+	-	+	+	-	-	-	-
DDX18/HAS1 subfamily	+	-	-	-	+	-	-	-	+	-
U1A snRNP	-	-	+	-	+	-	-	-	+	-
SmG	+	-	+	-	+	-	-	-	+	
Integrator complex subunit (Int11)	+	-	-	-	-	-	-	-	+	-
Zn finger protein	+	-	+		+	+	-	-	+	+
Regulator of nonsense transcripts 1 homolog (smg-2 like)	+	-	+	-	+	+	-	-	+	+
CaM Kinase	+	-	+	-	+	-	-	-	+	-
Serine/threonine p21-activated kinase (PAK) mbt like protein	+	-	+	-	+	-	-	-	+	-
cAMP-dependent protein kinase C1	+	-	+	-	+	-	-	-	+	-
Protein Kinase C	+	-	+	-	+	-	-	-	+	-
IKK-beta	+	-	+	-	+	-	-	-	+	-
STE20/Fray	-	-	+	-	+	-	-	-	-	-
MAPKK4	+	-	+	-	+	-	-	-	+	+
MDR1A	+	-	+	-	+	-	-	-	+	+
Tudor	+	+	+	-	+	+	-	-	+	+
Sil-2	+	+	+	+	-	-	+	+	+	+
Histone3 Lysine4 N-methyltransferase	-	-	-	-	+	+	-	-	+	+
Histone deacetylase 3 like	+	-	+	-	+	+	-	-	+	+
Gas41	+	-	+	-	+	-	-	-	-	-
eIF2B-gamma	+	-	+	-	+	-	-	-	+	+
eIF4AII	-	-	-	-	+	-	-	-	Homolog INF-1	-
eIF4AIII	+	-	+	-	+	-	-	-	+	-
RPL23A	+	-	+	-	+	-	-	-	+	+
KIF18A-like	+	-	+	-	-	-	-	-	+	-
Cyclin-dependent kinase 5 homolog	+	-	+	-	+	-	-	-	-	-
KIF3A-like	+	-	+	-	+	-	-	-	+	-
Isocitrate dehydrogenase	+	-	+	-	+	-	-	-	+	+
Myosin VIIa-like	+	-	+	-	+	-	-	-	+	-
Nucleolar complex protein 2 homolog	+	-	+	-	+	-	-	-	+	-
WD 40 like repeat domain	+	-	+	-	+	-	-	-	+	-
S-phase kinase-associated protein (SkpA)	+	-	+	-	+	-	-	-	+	-

The table shows a comparative analysis of identification and validation of candidate genes found in *Sf21* with those organisms for which genome wide approach has been performed in search for RNAi factors either by means of computational approach or functional genomics where (A) denotes *in sillico* identification of the putative candidate and (B) *in vivo* validation of the same as RNAi factor.

## Conclusions

To understand the dogma as well as the thematic variations of gene silencing, functional and comparative genomics serves as a great tool that attempts to describe gene functions and interactions of various components involved in RNA interference mechanism. The study illuminates the integration of small RNA related pathways with other basic cellular activities that govern gene expression for different cellular actions. The conservation of putative core RNAi factors found in insects highlights the biological characteristics of the insect phyla. Despite conservation the molecular evolution of the prime and accessory RNAi candidates of *Sf21* cells is quite appreciable. The significance of the unique RNAi factors of *Sf* cells needs to be assimilated and thus the new insights in the biology of this specific insect will be explored.

## Methods

### Annotation of RNAi factors from assembled genome and transcriptome data

To identify and characterize putative RNAi candidates in *S. frugiperda*, we considered predicted ORF’s from *Sf21* whole genome sequencing, mRNAs from whole transcriptome sequences and ESTs extracted from SPODOBASE [122] as well. A search for homologs of RNAi factors present in *Spodoptera frugiperda* genome was carried out using protein sequences of the known RNAi factors of *Bombyx mori*, *Tribolium* castaneum, *Drosophila melanogaster* and *C. elegans* using Blastx program. Query sequences over 70% coverage were carefully chosen as putative homologs of the corresponding RNAi genes in *S. frugiperda* genome followed by phylogenetic analysis, domain architecture and CDS generation of consensus of translated proteins [33] (SUB620801).

### siRNA designing

A total of eighty putative factors for RNAi were identified in *S. frugiperda* and for knockout analysis siRNAs for each of the gene were designed by Dharmacon Inc, siRNA designing tool. Multiple siRNAs were predicted for each candidate gene across the gene length with a varying score. In view of the varying *in vivo* efficacies of different siRNAs of the same gene, 3-5 siRNAs from different regions of the gene with high scores were selected. siRNA list of RNAi factors validated by functional assay with *gfp* reversion are summarized in Additional file 4.

### Cell culture and transfection

To perform siRNA knockdown assay in the *Sf21* cell line, a previously developed *Sf21* control line (constitutive *gfp* expressing cells in which *gfp* was integrated inside the *Sf*21 genome) was employed [30]. *Sf21* cells (Invitrogen) and *Sf21-gfp* reporter cell line were cultured in TNM-FH Insect Medium (BD BaculoGold) supplemented with Grace’s medium including trace metals, lactalbumin hydrolysate, yeastolate, and 10% heat inactivated fetal bovine serum along with 300 μg/ml Zeocin (100 mg/mL) (Invitrogen). One hour before transfection, cells (80–90% confluent) were sloughed and cell viability was determined by treating the cells with Trypan Blue. Cells with > 95% viability were uniformly transferred to 24-well plate containing BD Baculogold TNM-FH insect medium with cell density 2.5 × 10^5^/well and allow cells to attach. Prior to the addition of transfection mix, cells settled in the plate were washed three times with max-XP serum-free Insect cell medium (BD Baculogold). For reversion assay *Sf21-gfp* reporter cells were co-transfected with *gfp* siRNA (GGU UAU GUA CAG GAA CGC AUU) and test siRNA in the presence of Cellfectin II reagent (Invitrogen) incubated in 200 μl BD Baculogold max-XP serum-free medium for 20 minutes. The transfection mixture for *Sf21-gfp* reporter cells was prepared with *gfp* siRNA and Cellfectin II reagent which was treated as control *gfp* silenced cells. *Sf21*cells and *Sf21-gfp* reporter cells were also separately incubated only with serum-free medium. Besides, scrambled siRNA (UUG UCU UGC AUU CGA CUA AUdT) with *gfp* siRNA was used to transfect reporter cell line as negative control. Four hours after transfection, serum medium was added to the culture plate. 48 hours after transfection cells were processed for FACS analysis. All candidate siRNAs were retested in triplicate.

### Fluorescence microscopy and flow cytometric analysis

The level of *gfp* expression in the *Sf21-gfp* reporter cell lines and siRNA transfected cells were monitored using a Nikon Eclipse TE2000-U Fluorescence microscope (Nikon, Tokyo, Japan) followed by quantification with fluorescence activated cell sorting (FACS). For the FACS analysis, *Sf21* cells were washed with FACS-grade phosphate buffered saline (PBS; BD Biosciences) and re-suspended in 400 μl of FACS-grade PBS. To determine the *gfp* fluorescence of the cells FACSCalibur flow cytometry (Becton-Dickinson) was used. Fluorescence analysis was performed using BD CellQuest Pro Software (Becton-Dickinson). The % of the parent gate detecting *gfp* fluorescence of cell population for each set of transfection for the selected candidate genes has been listed in Additional file 3. From the FACS data, the following quantitative parameters were determined. These were (A) the number of *Sf21-gfp* expressing cells co-transfected with test siRNA and *gfp* siRNA, (B) the same for *Sf21-gfp* expressing cells transfected only with *gfp* siRNA, and (C) *Sf21-gfp* expressing cells. The % *gfp* reversion was calculated as {(A − B)/(C − B)} × 100.

### Validation and efficiency of siRNA transfection by real time PCR

Transfection efficiency of siRNAs was validated by performing a quantitative Real-Time PCR. Two sets of siRNAs of each of the putative candidates were transfected individually to *Sf21-gfp* cell line using standard protocol. 48 hours after transfection cells were proceed for RNA extraction using Trizol (Invitrogen). Quality and quantity of RNA was checked by using Nanodrop 2000 Spectrophotometer (Thermo Scientific). 100 ng of total RNA was quantified in a single step RT-qPCR assay with two biological replicates each in triplicates using Verso SYBR Green ROX Mix (Thermo Scientific) following manufacturer’s instructions in PIKOREAl 96 detection system (Thermo Scientific). Beta Actin was used as an endogenous control for RNA expression profiling. siRNA induced down regulation of selected genes were then calculated against *Sf21-gfp* cell line as a calibrator using 2^−ΔΔCT^ method. Relative expression and efficacy of siRNA induced knock down of six selected candidates (Dcr-1, Ago-1, Drosha, Loquacious, Tudor and Sil-2) were represented in Additional file 2. siRNAs used for knock down of selected genes were listed in Additional file 5 and Primers used for their amplification were listed in Additional file 6.

### Domain analysis

Domain architecture of proteins was analysed using NCBI conserved domain [123]. The query sequence was searched using NCBI Conserved Domains Database (CDD) v3.11-45746 PSSMs database. Only significant matches of each of the protein were taken where the bits score was greater than or equal to the gathering threshold for the Pfam domain.

### Phylogenetic analysis

Sequence manipulation and evolutionary analyses were conducted in MEGA5. Phylogenetic analyses were followed by CLUSTALW and using Neighbour-joining method with bootstrap test (1500 replicates) along with tree visualization was accomplished using MEGA 5 [124]. P-distance method was used to compute the evolutionary distance and was in the units of the number of amino acid differences per site. All positions containing gaps and missing data in amino acid sequences were eliminated.

### Search for putative RNAi factors orthologs

ORF of putative RNAi factors were used as the query in a Uniprot Blastx against arthropods, human and nematodes. Homology search was also crosschecked with Blastx against *Tribolium castaneum*, *Drosophila melanogaster*, *Bombyx mori*, *Caenorhabditis elegans*, *Homo sapiens* in the NCBI non-redundant protein sequence database to identify their respective orthologs. Most significant alignment with lowest E-value from each of the organisms were considered with the threshold of >50% in terms of either maximum identity or query coverage.

### Interactome analysis of *Sf21*RNAi factors

Known and Predicted Protein Interactions of *Tribolium* homologs of *Sf21* RNAi factors have been accumulated from STRING 9.1 database. Visualization of molecular interaction networks have been constructed using Cytoscape_v2.8.3 [125].

### Accession numbers

The Accession numbers used for identity search are summarized in Additional file 7. The nucleotide sequences that have been used to design siRNA reported in this study have been described in the manuscript [33] (SUB620801).

## Electronic supplementary material

Additional file 1: ***In silico*** **predicted gene list of eighty putative candidates for** ***Sf21*** **RNAi pathway.** (DOCX 28 KB)

Additional file 2: **Real-Time analysis to validate gene knockdown by measuring efficiency of siRNA of putative candidates.** (DOCX 18 KB)

Additional file 3: **Table containing %** ***gfp*** **expression obtained from post transfected FACS analysis in the functional assay for siRNA treated both** ***gfp*** **reverted and non-reverted genes.** (DOCX 21 KB)

Additional file 4: **List of siRNAs used for** ***gfp*** **reversion assay.** (DOCX 19 KB)

Additional file 5: **List of siRNAs used to validate transfection efficiency of selected genes.** (DOCX 15 KB)

Additional file 6: **List of primers used for qRT-PCR.** (DOCX 15 KB)

Additional file 7: **Accession numbers used for identity search of RNAi gene homologs with other insects.** (DOCX 20 KB)
