# Supplementary material for: Genome wide screening of RNAi factors of Sf21 cells reveal several novel pathway associated proteins
Source: BMC Genomics. 2014 Sep 9;15:775. doi: 10.1186/1471-2164-15-775 (PMC4247154; doi:10.1186/1471-2164-15-775)
Supplement: Supplementary file 1 — Additional file 1: In silico predicted gene list of eighty putative candidates for Sf21 RNAi pathway. (DOCX 28 KB) [file 12864_2014_6685_MOESM1_ESM.docx]

# Additional File 1

| **ORF Name** | |
| --- | --- |
|  | Argonaute-1 |
|  | Cleavage stimulation factor, 3′ pre-RNA subunit 2; 64 kDa |
|  | 60S ribosomal export protein NMD3-like |
|  | Dicer-1 |
|  | Aconitate hydratase, mitochondrial-like |
|  | S- phase kisase protein A |
|  | Multi drug resistance 49 (Mdr49) |
|  | RNA helicase Dbp45A subfamily |
|  | MAP kinase kinase 7 like |
|  | RNA helicase VASA subfamily |
|  | CENP-E Kinesin-like motor protein 3A like |
|  | WD 40 like repeat protein |
|  | Zinc finger protein |
|  | Dual specificity mitogen-activated protein kinase kinase 4 (MAP kinase kinase 4) (MAPKK 4) |
|  | Histone deacetylase 3 (HD3) |
|  | DNA-directed RNA polymerase III subunit RPC5-like |
|  | Gas 41 like factor |
|  | RNA helicase DDX18/HAS1 subfamily |
|  | Vigilin like |
|  | Isocitrate dehydrogenase [NAD] subunit alpha, mitochondrial like |
|  | RNA 3′-terminal phosphate cyclase-like protein |
|  | Myosin-XV -like |
|  | R2D2 |
|  | CENP-E Kinesin-like motor protein18A-like |
|  | Vacuolar ATP synthase subunit C |
|  | Cyclin dependent kinase 9 gene |
|  | DEAD box polypeptide 5 |
|  | PHD finger protein rhinoceros-like |
|  | Putative ATP-dependent RNA helicase DHX33-like |
|  | Putative pre-mRNA-splicing factor ATP-dependent RNA helicase DHX15-like |
|  | ATP-binding cassette, MDR1A |
|  | Drosha |
|  | pre-mRNA 3' end processing protein WDR33-like |
|  | DNA topoisomerase II |
|  | Isocitrate dehydrogenase [NAD] subunit beta, mitochondrial-like |
|  | Serine/threonine-protein kinase svkA-like |
|  | Myosin VIIa like |
|  | Serine/threonine-protein kinase TBK1-like |
|  | Translation initiation factor eIF-2B gamma subunit (eIF-2B GDP-GTP exchange factor) |
|  | Probable cleavage and polyadenylation specificity factor; Integrator complex subunit (Int11) |
|  | Dicer-2 |
|  | Serine/threonine protein phosphatase 2A, 65 kDa regulatory subunit A |
|  | Dual specificity mitogen-activated protein kinase kinase 6-like |
|  | Tudor |
|  | Serine/threonine p21 activated protein kinase (PAK) |
|  | Putative pre-mRNA-splicing factor ATP-dependent RNA helicase DHX16-like |
|  | Myeloid/lymphoid or mixed-lineage leukemia protein 3 homolog (Histone- lysine N-methyltransferase, H3 lysine-4 specific MLL3) |
|  | Calcium/Calmodulin dependent Kinase |
|  | Small nuclear Ribonucleoprotein G (SmG) |
|  | Casein kinase II beta subunit (CKIIbeta) |
|  | eIF4AIII |
|  | U1 small nuclear ribonucleoprotein A (U1 snRNP protein A) (U1-A) (Sex determination protein snf) |
|  | ATP-dependent RNA helicase DHX8-like |
|  | STE20/Fray like |
|  | Probable splicing factor, arginine/serine-rich 7-like |
|  | Regulator of nonsense transcripts 1 (Nonsense mRNA reducing factor 1); (smg-2 like) |
|  | Protein kinase C |
|  | Trimethylguanosine synthase-like |
|  | DEAD-box helicase Dbp80-like |
|  | Probable ATP-dependent RNA helicase DDX23-like |
|  | cAMP dependent protein kinase C1 |
|  | Histone deacetylase 8-like |
|  | Serine/threonine-protein kinase Warts-like |
|  | Ran GTP-binding Protein |
|  | Inhibitor of nuclear factor kappa-B kinase subunit beta (IKK-beta) |
|  | Myosin heavy chain, non-muscle-like |
|  | Nucleolar complex protein 2 homolog |
|  | Pasha |
|  | CENP-E Kinesin-like protein KIF23-like |
|  | Argonaute-3 |
|  | Loquacious |
|  | Sid-1 like protein (Sil-2) |
|  | Importin subunit alpha-7-like |
|  | Ribosomal subunit L23P |
|  | Histone deacetylase Rpd3-like |
|  | Aubergine |
|  | Brain tumor protein-like |
|  | Splicing factor 3A subunit 1 (Spliceosome associated protein 114) |
|  | Cyclin-dependent kinase 5 homolog |
|  | eIF4AII |
