# Supplementary material for: Genome wide screening of RNAi factors of Sf21 cells reveal several novel pathway associated proteins
Source: BMC Genomics. 2014 Sep 9;15:775. doi: 10.1186/1471-2164-15-775 (PMC4247154; doi:10.1186/1471-2164-15-775)
Supplement: Supplementary file 2 — Additional file 2: Real-Time analysis to validate gene knockdown by measuring efficiency of siRNA of putative candidates. (DOCX 18 KB) [file 12864_2014_6685_MOESM2_ESM.docx]

## Additional File 2

1. siRNA set 1
2. siRNA set 2
