# Supplementary material for: Genome wide screening of RNAi factors of Sf21 cells reveal several novel pathway associated proteins
Source: BMC Genomics. 2014 Sep 9;15:775. doi: 10.1186/1471-2164-15-775 (PMC4247154; doi:10.1186/1471-2164-15-775)
Supplement: Supplementary file 3 — Additional file 3: Table containing % gfp expression obtained from post transfected FACS analysis in the functional assay for siRNA treated both gfp reverted and non-reverted genes. (DOCX 21 KB) [file 12864_2014_6685_MOESM3_ESM.docx]

# Additional File 3

|  | **Quantification of *gfp* expression (% parent gate of FACS result)** | | | | | | | ***% gfp* Reversion** |
| --- | --- | --- | --- | --- | --- | --- | --- | --- |
|  | ***Sf21*** | ***Sf21-gfp*** | ***Sf21-gfp* + *gfp* siRNA** | ***Sf21-gfp* + *gfp* siRNA + test siRNA (1)** | ***Sf21-gfp* + *gfp* siRNA + test siRNA (2)** | ***Sf21-gfp* + *gfp* siRNA + test siRNA (3)** | ***Sf21-gfp* + *gfp* siRNA + scrambled siRNA** |  |
| **Core RNAi factors** | | | | | | | | |
| Dicer-2 | 0.37 | 72.29 | 44.02 | 55.26 | 56.35 * | 46.73 | 44.12 | 43.61 |
| Argonaute-1 | 0.37 | 72.29 | 44.02 | 51.66 | 57.76 * | 52.93 | 44.12 | 48.6 |
| Drosha | 0.94 | 38.56 | 16.47 | 24.49 | 25.3 * | 21.16 | 17.27 | 39.97 |
| Pasha | 0.06 | 42.24 | 19.94 | 25.52 | 24.70 | 30.08 * | 20.20 | 45.50 |
| Aubergine | 0.06 | 37.00 | 17.69 | 25.69 * | 21.09 | 21.42 | 17.88 | 41.42 |
| Loquacious | 0.77 | 61.15 | 49.26 | 48.51 | 48.63 | 51.23 * | 48.29 | 16.56 |
| **Auxilliary RNAi factors** | | | | | | | | |
| DDX18/HAS1 subfamily | 0.21 | 40.04 | 25.01 | 31.08 | 20.08 | 34.26 * | 25.57 | 61.54 |
| MDR1A | 0.9 | 45.60 | 15.14 | 29.18 | 31.75 | 35.70 * | 20.7 | 67.5 |
| Isocitrate dehydrogenase | 0.9 | 45.60 | 15.18 | 35.70 * | 31.46 | 14.40 | 16.74 | 67.4 |
| Tudor | 0.89 | 57.99 | 24.78 | 24.70 | 20.27 | 23.74 | 25.21 | - |
| Sil-2 | 0.89 | 64.07 | 24.78 | 28.41 * | 19.92 | 22.53 | 24.39 | 9.23 |
| **Putative RNAi factors with negligible/no reversion** | | | | | | | | |
| Serine/threonine protein phosphatase 2A | 0.54 | 44.59 | 24.22 | 24.66 | 24.28 | 25.43 * | 25.40 | 3.86 |
| Myosin-XV -like | 0.21 | 42.49 | 25.01 | 25.89 | 25.64 * | 24.39 | 24.55 | 3.60 |
| Splicing factor 3 subunit 1 | 0.25 | 51.5 | 24.76 | 25.6* | 20.62 | 24.44 | 24.96 | 3.14 |

Set of siRNA with % parent value of *gfp* expression marked as (*) has been considered for % *gfp* reversion calculation.
