# Supplementary material for: Genome wide screening of RNAi factors of Sf21 cells reveal several novel pathway associated proteins
Source: BMC Genomics. 2014 Sep 9;15:775. doi: 10.1186/1471-2164-15-775 (PMC4247154; doi:10.1186/1471-2164-15-775)
Supplement: Supplementary file 4 — Additional file 4: List of siRNAs used for gfp reversion assay. (DOCX 19 KB) [file 12864_2014_6685_MOESM4_ESM.docx]

# Additional File 4

| **Gene** | **siRNA sequences (5**′ **to 3**′**)** |
| --- | --- |
| Argonaute-1 | CGUGCAUGCGGUGACCAAATT |
| Argonaute-3 | CGGAAGAAGCAGAGCGTTTTT |
| Dicer-2 | CGAUAUAGAUUGGAGUGUATT |
| Dicer-1 | GATAAATCGTCCAAAGATATT |
| Aubergine | GTAAAGATGGCATTCATTA |
| Drosha | GAACUUUACCGUGAAAGAATT |
| Pasha | AGATGAAGAAACTGGAGTA |
| Loquacious | GCUUUGAAGAUAAACUGAUTT |
| R2D2 | AGGAAATGATGGTGAAGATTT |
| Dbp45A | GATCAAGTATTTAGTGCTT |
| VASA | CAACAGAGGCAAAGCAGTA |
| DDX18/HAS1 | GCGGAAAGACATTGGCATT |
| U1A snRNP | CGGCCAGATTTTGGACATT |
| SmG | AACUGAGCAUUAAACUGAATT |
| Integrator complex subunit (Int11) | GGACAATGAACTAGAGATT |
| Zn finger protein | CTTGGTGGAATGTGATGTA |
| Regulator of nonsense transcripts 1 homolog (smg-2) | GACCUUUCUGGAUACCGAATT |
| CaM Kinase | GGATGATATTAGTGATTCA |
| Serine/threonine p21-activated kinase (PAK) mbt like protein | CGGGTGAAATTATCTGATT |
| cAMP-dependent protein kinase C1 | GAGCATACGTTAAATGAAA |
| Protein Kinase C | CTGATAACAGCGTGTCTTA |
| IKK-beta | GGACGAAGGAAGTGGAAAT |
| STE20/Fray | GGACAGATACACAGAGATA |
| MAPKK4 | CCACCGAGGCTAACCAATA |
| MDR1A | TGAAGCGAAAGTACAGAAA |
| Sil-2 | CCAGAUGGAUGAAAACGAATT |
| Histone3 Lysine4 N-methyltransferase | GACCAAGAATTCCAACCTA |
| Histone deacetylase 3 like | TGAAATTGGGGCTGAAAGT |
| Gas41 | CAAAGACGAAATTGCGAAA |
| eIF2B-gamma | GAAAGGAACCTCAGAGAAA |
| eIF4AII | GCTGGAGAGTGGTGTGCAT |
| eIF4AIII | GUUUCAAAGAGCAGAUCUAUU |
| RPL23P | GCGAAAGTTAACACCCTTA |
| KIF18A-like | CCTCTGAACCAAAGGGAAC |
| Cyclin-dependent kinase 5 homolog | CCCAGAACTTGTTGATAAA |
| KIF3A-like | CCATATTGCCAAAGCTAAT |
| Isocitrate dehydrogenase | GTAGTGACAATCAGAGAGA |
| Myosin VIIa-like | AGTCTAGAATAGTCTCTCA |
| Nucleolar complex protein 2 homolog | GTGTTGAGATTCTGATAAA |
| WD 40 like repeat domain | GTGTACAATTTAACCCCAA |
| S-phase kinase-associated protein (SkpA) | CCAATATGATTAAGGGTAA |
