# Supplementary material for: Genome wide screening of RNAi factors of Sf21 cells reveal several novel pathway associated proteins
Source: BMC Genomics. 2014 Sep 9;15:775. doi: 10.1186/1471-2164-15-775 (PMC4247154; doi:10.1186/1471-2164-15-775)
Supplement: Supplementary file 5 — Additional file 5: List of siRNAs used to validate transfection efficiency of selected genes. (DOCX 15 KB) [file 12864_2014_6685_MOESM5_ESM.docx]

# **Additional** File 5

| **siRNA marked as** | **siRNA sequences (5**′ **to 3**′**)** |
| --- | --- |
| Dicer-1 (1) | CGAAAGAGAUAGAUGAUAAUU |
| Dicer-1 (2) | GAU AAAUCGUCCAAAGAUAUU |
| Argonaute-1 (1) | CGUGCAUGCGGUGACCAAATT |
| Argonaute-1 (2) | CGAUCUGUAUGUGAAAGAATT |
| Drosha (1) | GAACUUUACCGUGAAAGAATT |
| Drosha (2) | CGAUAUGCAGAAAAUUAAATT |
| Loquacious (1) | GCUUUGAAGAUAAACUGAUTT |
| Loquacious (2) | GGAUCUGAAAGAUAGCAAATT |
| Tudor (1) | AAACCGAAAAAGAACGCAATT |
| Tudor (2) | GCAAAAACCUGAUUAAAGATT |
| Sil-2 (1) | CCAGAUGGAUGAAAACGAATT |
| Sil-2 (2) | CCCGGUGUUUGAUAACGAATT |
