# Supplementary material for: Genome wide screening of RNAi factors of Sf21 cells reveal several novel pathway associated proteins
Source: BMC Genomics. 2014 Sep 9;15:775. doi: 10.1186/1471-2164-15-775 (PMC4247154; doi:10.1186/1471-2164-15-775)
Supplement: Supplementary file 6 — Additional file 6: List of primers used for qRT-PCR. (DOCX 15 KB) [file 12864_2014_6685_MOESM6_ESM.docx]

# Additional File 6

| Gene | Primer Sequence (5’ to 3’) |
| --- | --- |
| Dicer-1 | For: CTGAAGGGGAGTTGGAACAA  Rev: GGAACTCGAGTCGCTGGTAG |
| Argonaute-1 | For: ATCAAGGAATTCAAGGCACG  Rev: GAGTCGTGCTCCTTCTCCAC |
| Drosha | For: GCGGAATGCTAGACCTGAAG  Rev: GGTGACTCTGCTTCCTTTGC |
| Loquacious | For: GCTCCGGCTGATCAGTCTAC  Rev: CGGCAAACGCCTATTAACAT |
| Tudor | For: CCTTCGGTGAAGAAGCTTTG  Rev: GCAAACTCTGAAGTTTCCGC |
| Sil-2 | For: TGACGTTTCAACCCAGAACA  Rev: GACGAAAAAGTCTTGGACGG |
