# Supplementary material for: Genome wide screening of RNAi factors of Sf21 cells reveal several novel pathway associated proteins
Source: BMC Genomics. 2014 Sep 9;15:775. doi: 10.1186/1471-2164-15-775 (PMC4247154; doi:10.1186/1471-2164-15-775)
Supplement: Supplementary file 7 — Additional file 7: Accession numbers used for identity search of RNAi gene homologs with other insects. (DOCX 20 KB) [file 12864_2014_6685_MOESM7_ESM.docx]

# Additional File 7

| **Gene** | **Bm Ac No.** | **Tc Ac No.** | **Dm Ac No.** |
| --- | --- | --- | --- |
| Argonaute-1 | A7BJS5 | D6WVW4 | Q32KD4 |
| Argonaute-3 | BAF98575.1 | XP_968053.2 | ABO27430.1 |
| Dicer-2 | D7UT11 | NP_001107840 | A1ZAW0 |
| Dicer-1 | XP_004922366.1 | EFA11550.1 | ABD61602.1 |
| Aubergine | NP_001098066 | XP_001811159 | AGA18946 |
| Drosha | XP_004928266.1 | XP_967454.2 | NP_477436.1 |
| Pasha | XP_004922769.1 | XP_971282.1 | NP_651879.1 |
| Loquacious | D7UT12 | D6X0T5 | Q9VJY9 |
| R2D2 | NP_001182007.1 | NP_001128425.1 | ABB77175.1 |
| Dbp45A | H9JID6 | D2A1T9 | Q07886 |
| VASA | H9J3E9 | D6WSI7 | P09052 |
| DDX18/HAS1 | XP_004930677.1 | XP_973872.2 | NP_732694.2 |
| U1A snRNP | NP_001037384.1 | XP_968271.1 | NP_511045.1 |
| SmG | NP_001040405.1 | XP_967705.1 | NP_573139.1 |
| Integrator complex subunit (Int11) | XP_004928171.1 | NP_651721.1 | XP_969343.1 |
| Zn finger protein | H9ISS7 | D7EJN8 | Q9VBB3 |
| Regulator of nonsense transcripts 1 homolog | XP_004929661.1 | XP_973320.2 | NP_572767.1 |
| CaM Kinase | XP_004929500.1 | XP_967941.2 | NP_524622.1 |
| Serine/threonine p21-activated kinase (PAK) mbt like protein | XP_004928420.1 | XP_969620.2 | AAK93447.1 |
| cAMP-dependent protein kinase C1 | NP_001093303.1 | XP_968170.1 | NP_476977.1 |
| Protein Kinase C | Q4AED6 | D6WG72 | D3DMK5 |
| IKK-beta | XP_004928169.1 | XP_969880.1 | AAG02485.1 |
| STE20/Fray | XP_004930020.1 | XP_974782.1 | AAD01275.1 |
| MAPKK4 | XP_004927053.1 | XP_974082.1 | AAC39036.1 |
| MDR1A | XP_004924686.1 | XP_001810982.1 | NP_523740.3 |
| Tudor | NP_001182009.1 | XP_974879.1 | NP_612021.1 |
| Sil-2 | A9CQL3 | A7YFW2 |  |
| Histone3 Lysine4 N-methyltransferase | H9IZ17 | D6W9D4 | Q8IRW8 |
| Histone deacetylase 3 like | XP_004922748.1 | XP_969419.1 | NP_651978.2 |
| Gas41 | XP_004930356.1 | XP_973381.1 | NP_609086.1 |
| eIF2B-gamma | NP_001037654.1 | XP_971487.1 | NP_611046.2 |
| eIF4AII | ABF51379.1 | NP_001177648.1 | NP_476595.1 |
| eIF4AIII | NP_001106217.1 | XP_975511.1 | NP_649788.2 |
| RPL23P | NP_001037229.1 | XP_967229.1 | NP_523886.1 |
| KIF18A-like | XP_004924749.1 | XP_967885.1 | AAB49460.1 |
| Cyclin-dependent kinase 5 homolog | NP_001162053.1 | EFA06801.1 | NP_477080.1 |
| KIF3A-like | XP_004926473.1 | XP_971025.2 | NP_523934.1 |
| Isocitrate dehydrogenase | XP_004925062.1 | XP_970030.2 | NP_001259705.1 |
| Myosin VIIa-like | XP_004931052.1 | XP_975112.2 | AAR96124.1 |
| Nucleolar complex protein 2 homolog | XP_004930956.1 | XP_968623.1 | NP_610095.1 |
| WD 40 like repeat domain | NP_001040226.1 | XP_966556.1 | NP_609217.1 |
| S-phase kinase-associated protein (SkpA) | NP_001040518.1 | XP_974524.1 | NP_477390.1 |
